# The Naïve Bayes classifier++ for metagenomic taxonomic classification—query evaluation

**DOI:** 10.1093/bioinformatics/btae743

**Published:** 2024-12-19

**Authors:** Haozhe (Neil) Duan, Gavin Hearne, Robi Polikar, Gail L Rosen

**Affiliations:** Ecological and Evolutionary Signal Processing and Informatics (EESI) Laboratory, Drexel University, Philadelphia, PA 19104, United States; Ecological and Evolutionary Signal Processing and Informatics (EESI) Laboratory, Drexel University, Philadelphia, PA 19104, United States; Signal Processing and Pattern Recognition Laboratory, Electrical and Computer Engineering, Rowan University, Glassboro, NJ 08018, United States; Ecological and Evolutionary Signal Processing and Informatics (EESI) Laboratory, Drexel University, Philadelphia, PA 19104, United States

## Abstract

**Motivation:**

This study examines the query performance of the NBC++ (Incremental Naive Bayes Classifier) program for variations in canonicality, k-mer size, databases, and input sample data size. We demonstrate that both NBC++ and Kraken2 are influenced by database depth, with macro measures improving as depth increases. However, fully capturing the diversity of life, especially viruses, remains a challenge.

**Results:**

NBC++ can competitively profile the superkingdom content of metagenomic samples using a small training database. NBC++ spends less time training and can use a fraction of the memory than Kraken2 but at the cost of long querying time. Major NBC++ enhancements include accommodating canonical k-mer storage (leading to significant storage savings) and adaptable and optimized memory allocation that accelerates query analysis and enables the software to be run on nearly any system. Additionally, the output now includes log-likelihood values for each training genome, providing users with valuable confidence information.

**Availability and implementation:**

Source code and Dockerfile are available at http://github.com/EESI/Naive_Bayes.

## 1 Introduction

The näive Bayes classifier (NBC) has proven itself to be a useful tool for classifying reads from amplicon and metagenomic samples (Wang *et al.* 2007, Rosen *et al.* 2008, 2011, Lan *et al.* 2012, Zhao *et al.* 2020). For 16S rRNA taxonomic classification, it is the gold standard (Ziemski *et al.* 2021) due to its superior ability to memorize k-mers unique to different taxa. For metagenomics, NBC is successful in determining the most relevant taxa and their abundances in most samples (despite using a limited database compared to others) (McIntyre *et al.* 2017).

While NBC is computationally tractable for 16S rRNA due to its simplicity, NBC is not more widely used in metagenomics because of scalability issues (McIntyre *et al.* 2017), as well as the difficulty of providing confidence in its predictions without significantly increasing computational time (Lan *et al.* 2012). In fact, taxonomic classifier speed can vary by 500-fold for short reads (Ye *et al.* 2019) and 80-fold for long reads (Marić *et al.* 2024), depending on accuracy of the results, presenting a performance-speed tradeoff. Also, there is a precision-recall trade-off. Kraken2/Bracken/Centrifuge assign the most number of reads to species but as a result have lower F1 score for fidelity of the assignments (Portik *et al.* 2022). Conversely, mOTUs and Sourmash are more conservative about their assignments but have good precision/recall values (Portik *et al.* 2022). There are two specific NBC scalability issues: (i) one issue is that k-mers must be counted during training, which is time-consuming, and their frequencies must be calculated (Zhao *et al.* 2020). In Zhao *et al.* (2020), we addressed this issue, by adding a capability of adding genomes to the database without having to recompute all k-mer frequencies of the entire database. (ii) Another issue is in the testing process of evaluating queries. In the previous implementation of NBC++ (Zhao *et al.* 2020), k-mer counting of the queries was conducted utilizing Jellyfish (Marçais and Kingsford 2011) and classes were added incrementally to the database. Due to Jellyfish requiring a file for each separate count, this approach often resulted in the creation of an extensive number of files, frequently reaching hundreds of thousands or even millions for a single metagenomic sample. To mitigate this challenge, the current study introduces a novel runtime counting mechanism within the testing module to reduce disk I/O.

Here, we examine a variety of parameters that offer speedups (sometimes at the cost of recall/precision), such as k-mer size and canonical versus non-canonical k-mer counting. We also experiment with three different database sizes to show the speed versus accuracy trade-off of using small but diverse datasets versus large, rich databases. Kraken2 (Wood *et al.* 2019) and Ganon2 (Piro and Reinert 2024) are chosen for comparison due to their similar functions in classifying all reads [and not solely compositional analysis (Portik *et al.* 2022)] and because of their competitive performance (Meyer *et al.* 2022). CAMI2 demonstrated that Kraken v.2.0.8 beta had the best accuracy on taxonomically binning contigs, and Ganon was best for short reads (Meyer *et al.* 2022). Finally, we evaluate how long it takes for NBC++ to classify a large-scale real human metagenomic sample when trained on increasing database depths.

## 2 Improvements/methodology since Zhao *et al.*

The aim of our previous research was to augment the size of the training database and enable incremental database updates (Zhao *et al.* 2020). In this article, we shift our focus from the training process to introduce a suite of enhancements aimed at refining the querying mechanism. These improvements are articulated as follows:

1. We optimized memory allocation within the confines of existing memory limitations, thus ensuring a more effective deployment of computational resources and adherence to memory constraints.
2. We introduce new features that facilitate the generation of full log-likelihood outputs, enabling a more thorough examination of the result distribution by users.
3. We implement canonical counting techniques which have led to a notable reduction in both the volume of training data and the disk space required for storing k-mer counts, bolstering the system’s overall efficiency.
4. We restructure the querying operational protocol to decrease frequent loading of training data from disk (when possible—the user must specify a relatively large memory limit), which in turn mitigates computational overhead and enhances the efficiency of the querying process (see the Appendix for more details about on-the-fly k-mer counting to reduce I/O).

### 2.1 Allocation of memory

The querying part of NBC++ has been improved to fit into memory specified by the user and can adapt to almost any memory size (a minimum of 2 GB is suggested). Such processing is in contrast to Kraken2, which takes up to 9 GB for classifying against the Basic database, 107 GB for the Standard database, and 206 GB for the Extended database (Ganon2 requires 1.5 GB, 66 GB, and 146 GB, respectively); see a description of these data under Section 2.4. Most of our experiments were performed with 180 GB of memory; however, we have also benchmarked a 64 GB memory/8-core comparison (as a desktop example in Section 3.1.5) demonstrating that computational time decreases linearly with the amount of concurrent cores (and computational time does not increase much when memory is reduced—30% longer runtime when provided 3× less memory).

### 2.2 Setting capacities

In the NBC++ system, each processing thread is allocated:

- a workload capacity of 1000 query reads.
- an output buffer capacity of 1000 entries.

These capacities are the result of optimization on Illumina simulated data for minimizing read and write delays.

See the Supplementary Material Appendix on *Estimating Memory Costs* for (i) how we load in trained classes and batches of query reads and (ii) equations on how the input and output buffers of the querying process are reserved and memory is utilized.

### 2.3 Log-likelihood score outputs

There are now two modes of log-likelihood outputs. The default output produces the **max** log-likelihood score and the taxa corresponding to the **max** log-likelihood score, followed by the lineage of that taxa (upper taxonomic levels). We also implemented a feature so that users can explore the log-likelihood values of each query against each genome in the database, with the -f flag. This verbose output will increase the output tabular file greatly, resulting in each query’s log-likelihood score against each genome in the database and not just the max. The user will likely need to adjust the -r and -c options to output reasonably sized tabular outputs. (The defaults are approximately the limits of what Excel can ingest.)

### 2.4 Data collection and database construction

This study uses the Refseq retrieval pipeline developed as a part of the Woltka software (Zhu *et al.* 2022) to sample the NCBI RefSeq database at high microbiome diversity at varying degrees of depth, and then test the performance of NBC++ on the resulting databases.

####  

We used Woltka’s refseq_build.py script to extract genomic data from the RefSeq database. This script was employed to create databases with distinct profiles:

- “Basic” database: comprising one genome per genus (with exceptions of Baxterfoxvirus and Betanucleorhabdovirus) resulting in a compilation of 4634 genomes as of 24 July 2023.
- “Standard” database: encompassing all NCBI-defined reference and representative genomes, totaling 18 237 genomes collected on 26 July 2023.
- “Extended” database: featuring one genome per species with a Latinate name and higher ranks, accumulating 319 554 genomes by 26 July 2023. The extended database also includes reference, representative, and type material genomes. The genomes were then grouped by species resulting in: **4634 classes** for “Basic,” **18 219 classes** for “Standard,” and **58 978 classes** for “Extended” (see Supplementary assembly files*_assemblysummary.txt, where * is basic/standard/extended).

### 2.5 Data sources and experimental design

*Simulated data*: For all simulated experiments, we used a 5-fold cross-validation framework. First, the dataset comprising various classes was randomly shuffled. Subsequently, we set the first one-fifth of the shuffled array as the test set for the first fold, with the remaining data serving as the corresponding training set. We repeated this procedure five times, using a different one-fifth segment (fold) as the test set, to generate the 5 folds. For the generation of testing reads, we used InSilicoSeq (Gourlé *et al.* 2018) to synthesize 100 reads per class, utilizing the metagenomic file that was sampled from the RefSeq database.

*Real human gut metagenomic sample*: To assess a real sample, we again use the data from human gut sample analyzed in Nasko *et al.* (2018) and Zhao *et al.* (2020). With such a sample, we can assess the composition of the sample with relative abundances and compare against various parameters with the Bray-Curtis distance (Zhao *et al.* 2020).

## 3 Results

We benchmark k-mer size, canonicality, and database depth on the querying results and time. For training, the number of k-mers is counted and either stored canonically or non-canonically. Since add-1 (Laplacian) smoothing is used for estimating the probability of the $i_{th}$k-mer in a genome as $P(k{\text{mer}}_{i}|G)=\frac{\mathrm{fre}q_{G}(k{\text{mer}}_{i})}{N_{G}+V}$ where freq${}_{G}$(k-mer${}_{i}$) is the ith k-mer in genome G, $N_{G}$ is the number of k-mers in genome G, and V is the vocabulary size of all k-mers in all genomes. V can be approximated at $V=4^{k}$ whenever a k-mer occurs at least once in one of the genomes in the entire database. For canonical k-mers, for k-mers of even length, the formula becomes $V=\frac{1}{2}(4^{k}+4^{k/2})$, while for those of odd length, it simplifies to $V=\frac{1}{2}4^{k}$ (Wittler 2023). These estimates are then used in the maximum likelihood formulation of NBC++ (Rosen *et al.* 2008).

### 3.1 Parameter sweeps

#### 3.1.1 Canonical versus non-canonical

In the present update, we switched to employing canonical representations for the enumeration and categorization of sequences, thereby achieving a reduction in computational time and memory allocation for resultant savefiles. Canonical counting chooses between the k-mer in question and its reverse complement, with the lexicographically smaller sequence being selected for utilization. Conversely, non-canonical counting involves the direct application of the k-mer as it stands. As illustrated in Supplementary Fig. S1, canonical and non-canonical counting exhibit comparable efficacy in the context of Basic 9-mer and Standard 9-mer. While there is little querying time speedup with the canonical representation, the amount of disk space saved can vary. For example, with 9-mers, where almost every 9-mer exists in long genomes, the canonical count representation uses 54% of the disk space (compared to the non-canonical representation). For 15-mers, the disk savings diminish—with long microbes still taking up to 90% and the longest fungi taking 83% of the non-canonical genome k-mer count file size.

#### 3.1.2 Varying the K-mer size with diverse database

We assess the performance and run-time of the classifier using 100 reads per genome in a random 5-fold cross-validation experiment against the Standard database.

Supplementary Figure S2 demonstrates the impact of varying k-mer sizes on the performance of the “Standard” database across different taxonomic levels. A noticeable trend is the steady increase in recall for lower taxonomic classes, specifically “family” and “genus,” with increasing k-mer sizes. Such an observation suggests that the model becomes more adept at distinguishing between finer, more related taxa as it utilizes longer k-mers. As we show in Supplementary Fig. S3, longer k-mers come at a cost of longer computational time, especially in training but also in testing.

Unexpectedly, the graph indicates a huge drop in performance at higher taxonomic levels, specifically between 9- and 12-mers for the superkingdom level. The rate of drop in recall decreases for 14–15 mers. The drop in recall rates for the 9–12 mers is a point of interest as it suggests a complex interplay between k-mer size and classification accuracy.

The complex interplay of k-mers relates to long- and short-range evolution. The database, composed mostly of prokaryotes known for high mutation rates (as compared to eukaryotes), produces results that shows that shorter k-mers are effective at capturing higher levels of taxonomic classification of the reads, similar to the effectiveness of tetramers in similar scenarios (Teeling *et al.* 2004). Longer k-mers, while capturing specific genomic details (e.g. mutations) of recent evolution, are susceptible to mutations caused by distant evolution, resulting in “class specificity.” Shorter k-mers are more likely to be less class specific and pick up higher level taxonomic signals. (Please also see Supplementary Appendix for a discussion of the effect of smoothing favoring long genomes.)

As indicated in the superkingdom confusion matrix in Supplementary Fig. S5a, we can see that recall rates (for small k-mer sizes) are high for Bacteria—but low for phyla in Eukaryotes/Viruses/Archaea. At large k, recall rates improve for Eukaryotes, but drop for Bacteria. Recall may improve for Eukaryotes because of the fact that Eukaryotes have longer k-mers that are more distinctive while prokaryotes have more mutational disruptions. NBC++ tends to be biased toward certain classes, which is reflected in the micro versus macro recall averages (Supplementary Figs S2 and S4). Micro-precision/recall are measures to calculate overall precision/recall which do not correct for the imbalance of classes (e.g. when simulating from the Standard database, more prokaryotes are simulated resulting in an imbalance of instances from kingdoms). In macro-precision/recall, the precision/recall is calculated for each class, and then it is averaged over the classes via the arithmetic mean to obtain the macro measure. Therefore, macro-precision/recall are class-balanced measures. NBC++’s lower performance for macro-recall rather micro-recall demonstrates that NBC++ tends to call some classes with higher recall than others (usually biased toward well-represented classes). Since NBC++ labels all reads (and does not leave reads unalabeled if they have low confidence), false positives for a predicted class are the same as false negatives for a true class. Therefore, micro-recall and micro-precision are synonymous for NBC++. Additional detail is available in the confusion matrices for common human gut taxa in Supplementary Fig. S5a–f.

#### 3.1.3 K-mer sweeps for prokaryotic kingdoms

The observations of surprisingly low classification performance (compared to our previous works) led to our hypothesis that the inclusion of the full diversity of life, particularly when considering the marked distinctions between Bacteria and Viruses, might introduce inconsistencies in classification performance. To test this assumption, the experimental procedure was replicated with a modification: the training set was exclusively composed of prokaryotic sequences.

The results from training on prokaryotic-only sequences are presented in Supplementary Fig. S6a–c, which reveal an overall positive micro-recall trend across all taxonomic levels in relation to the increasing lengths of k-mers. This observation is similar to previous behavior, albeit at lower performance due to the higher diversity of the database. The superkingdom micro-performance is similar to what we would expect [similar to accuracy rates observed in Zhao *et al.* (2020)], and the discerning classifications are further seen in the macro-recall/precision.

#### 3.1.4 Varying the database size

We constructed and generated testing reads for the three distinct database configurations: Basic, Standard, and Extended. Subsequent classification tasks were carried out, wherein the testing reads of each database were evaluated against their respective training sets. The results of these experiments are illustrated in Supplementary Fig. S7a–c, which highlight the performance dynamics across these database variations.

A notable observation from the micro-recall/precison graph in Supplementary Fig. S7a is the substantial improvement in classification performance when transitioning from the Basic to the Standard database configuration. Because of the heavy representation of prokaryotes in the training and testing sets of the Standard database, and since the classifier is better at identifying prokaryotes, micro-recall is high at the superkingdom level due to this bias in the Standard database. The macro-recall graph (Supplementary Fig. S7b) shows that recall improves when using Standard database (compared to the Basic database). Furthermore, we observe very little improvement in macro-precision (Supplementary Fig. S7c) with the Standard database, which itself improved with the use of the Extended database. These observations show that NBC++ performs better at superkingdom level, but its performance drops off for finer taxonomic levels.

#### 3.1.5 Query input data size versus time

To validate the linear computational time of NBC++, we conducted an experiment to monitor the runtime of the algorithm while increasing the amount of data. This experiment fixed the k-mer size at 9 and used the Standard database for classification. The computational resources allocated for this experiment included a fixed system with 180 GB of RAM and 48 processing cores. The results, depicted in Supplementary Fig. S8, affirm that the classification time scales linearly with the size of the input file.

We further evaluated the classification task on a typical personal computer, characterized by 64 GB of memory and 8 processing cores. Classifying an input query file of 240 MB, using 9-mers against Standard database, took 47.3 h, approximately 6.3× longer than 180 GB/48-core runs, again showing that the main factor affecting wall-clock time is the linear relation to multi-threading, and that there is a slight overhead due to memory partitioning.

### 3.2 Kraken2 results for comparison to NBC++

For all datasets, genomes were first added to a Kraken2 library sequentially (to decrease the wallclock time of this step, the genomes in extended folds 2–5 were added through five parallel processes; see Kraken2 add-to-lib in the Supplementary Appendix for more details). Then, a custom Kraken2 database was built using default settings for each fold. To minimize the number of unclassified reads in testing, the confidence parameter (—confidence) was set to 0.001, a low enough value to result in classifications with just a single k-mer match to a reference sequence.

Given that NBC++ performance was significantly lower for a diverse database of Prokaryotes, Eukaryotes, and Viruses, we wanted to evaluate how two of the best short-read classifiers, Kraken2 and Ganon2, would perform with the same training databases (see Supplementary Fig. S9a–d). It is important to note with Kraken2/Ganon2 that the micro-precision measure becomes different than micro-recall, since Kraken2/Ganon2 filter out many of the reads as “unclassified.” In the micro-recall/precision analysis, unclassified reads are considered as false negatives and are never considered as false positives (therefore micro-precision diverges from micro-recall). Kraken2/Ganon2 achieve high precision performance; however, their recall performances are poor. On the other hand, Kraken2 struggles with a diverse database, and only achieves a little over 30% in macro-recall for the Extended database and 10% or less with the Basic database (with better precision and similar recall performance to that of NBC++). Ganon2 is competitive on the superkingdom level for the micro-measures but is substandard to Kraken2 for all other levels and macro-measures. Kraken2 versus NBC++ comparison results can be found in Supplementary Fig. 10a and b, where we observe that Kraken2 exhibits superior performance for macro-measures (except for the superkingdom level for prokaryotes), particularly as the database size expands.

Regarding time efficiency of NBC++ and Kraken2 classifiers, Supplementary Fig. S11 provides an overview of the CPU time for a complete run, encompassing both the training and classification phases. We observe that NBC++, when employing a 9-mer approach, demands significantly less training time compared to Kraken2. However, the majority of Kraken2’s time expenditure is allocated to the database construction phase. Once the database is established, Kraken2 demonstrates remarkable speed in the classification of new input queries, outpacing NBC++ in this respect. This delineation suggests that while NBC++ is efficient in a holistic sense, Kraken2’s architecture allows it to excel in rapid query classification following the initial database setup.

### 3.3 Human gut sample classification versus database depth

**Figure 1.**
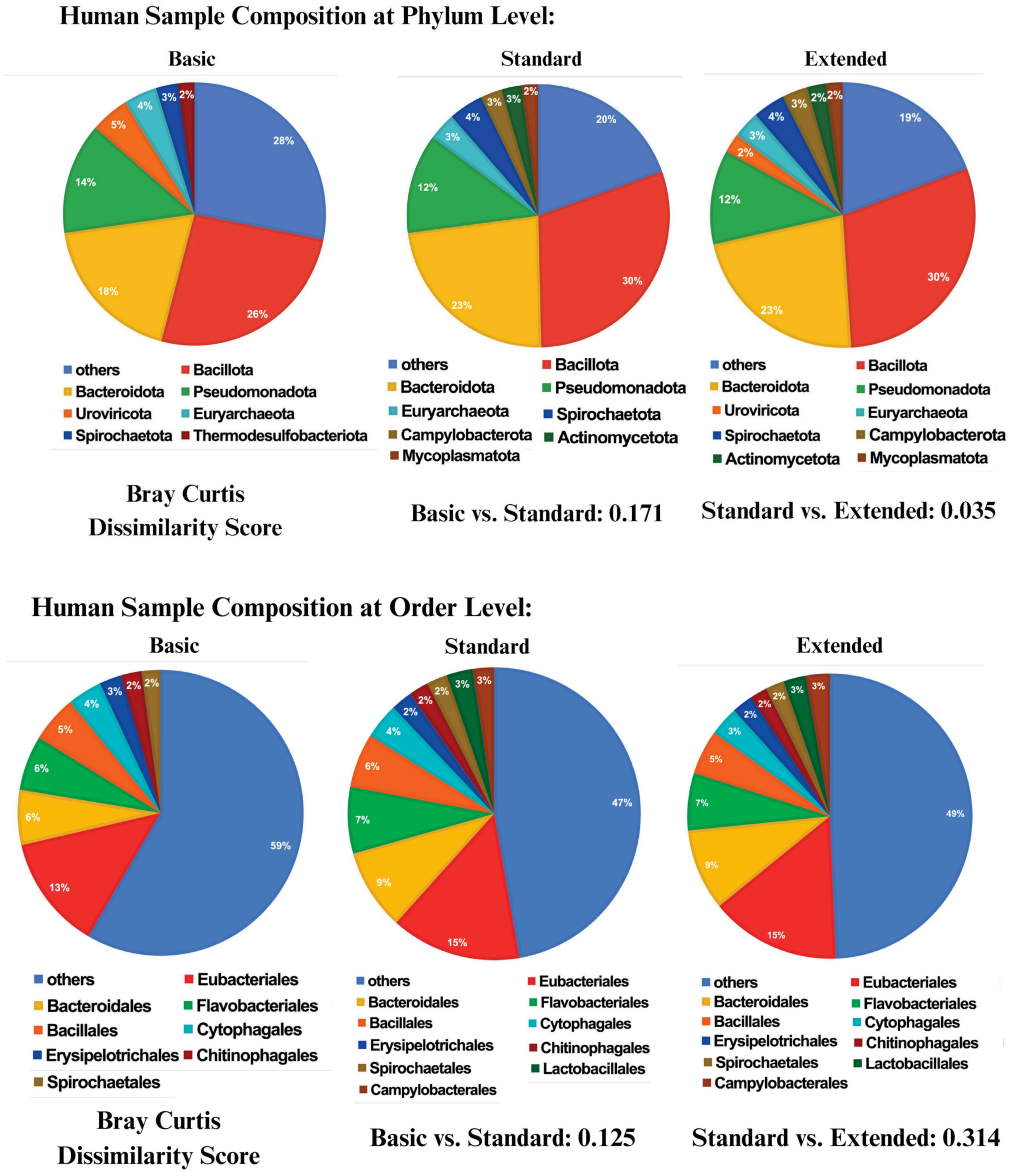
The Phylum and Order (over 2% relative abundance) composition of the human sample is shown. Standard and Extended databases have more concordance than Basic and Standard databases.

To analyze a human gut sample, we used k = 9, and examined the results across the three database depths (Basic, Standard, and Extended), shown in Fig. 1 and Supplementary Fig. S12. Fig. 1 also shows the Bray–Curtis dissimilarity metric to gauge the comparative similarity between the classified compositions of the human gut sample across the different databases. We see that Standard and Extended databases have more concordance than Basic and Standard databases, which is expected due to the sparse representation of the Basic database. We note that the Standard database seems to lack good representation of viruses, as shown for the superkingdom analysis (Supplementary Fig. S12a). Uroviricota are found in significant quantities in both Basic and Extended databases, with Basic indicating 3% more relative abundance than Extended. Because of few viral labels at the order level, Standard and Extended databases are very similar.

## 4 Conclusion

NBC++ can now be queried with realistic, large datasets and provide reasonable classification with a minimal database, especially in identifying superkingdoms and phyla. NBC++ requires less training time and much less memory than Kraken2, but at a cost of more testing (inference) time. While we used the most competitive short-read taxonomic classifiers for comparison, conclusions are limited due to the lack of comprehensive comparisons.

We tested diverse databases of at least one example per genera (Basic), one (or a few) per species (Standard), and more examples per species (Extended). While it was thought that such a database would represent the tree of life, we found that even a state-of-the-art classifier struggled with training on prokaryotes, viruses, and eukaryotes at the species level. Hopefully, the study within the paper provides a jumping point for further studies to examine the effect of breadth and depth of databases on taxonomic classifiers.

We should also note that an important improvement is the ability to obtain all log-likelihoods (of each read against each database genome). This ability enables future work of implementing a confidence measure and/or novelty detection using the log-likelihood scores determined by NBC++.

## Supplementary Material

btae743_Supplementary_Data

## Data Availability

NBC++ source code is available at http://github.com/EESI/Naive_Bayes. A docker container is available at https://hub.docker.com/r/eesilab/nbc_complete_toolset. The trained classifier.dat files for canonical 9-mers for Basic/Standard/Extended databases and the database-associated assembly summary files are available at https://zenodo.org/records/11657719. Summarized data results are at https://zenodo.org/records/11643985. The human gutreads are already assigned SRA ID: SRS105153.
