# Supplementary material for: The Naïve Bayes classifier++ for metagenomic taxonomic classification—query evaluation
Source: Bioinformatics. 2024 Dec 19;41(1):btae743. doi: 10.1093/bioinformatics/btae743 (PMC11729721; doi:10.1093/bioinformatics/btae743)
Supplement: btae743_Supplementary_Data [file btae743_supplementary_data.zip › supplementary_figures.pdf]

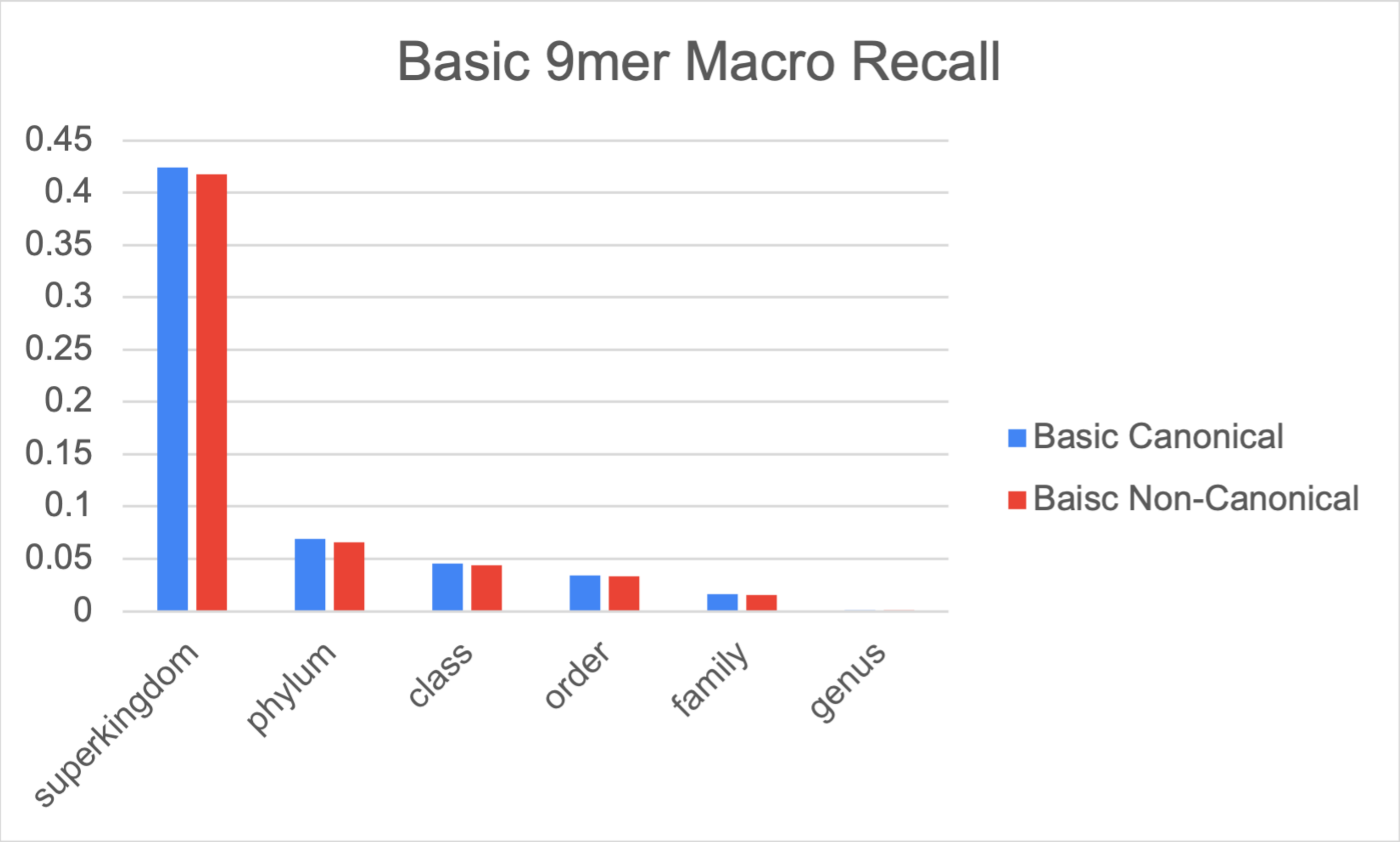

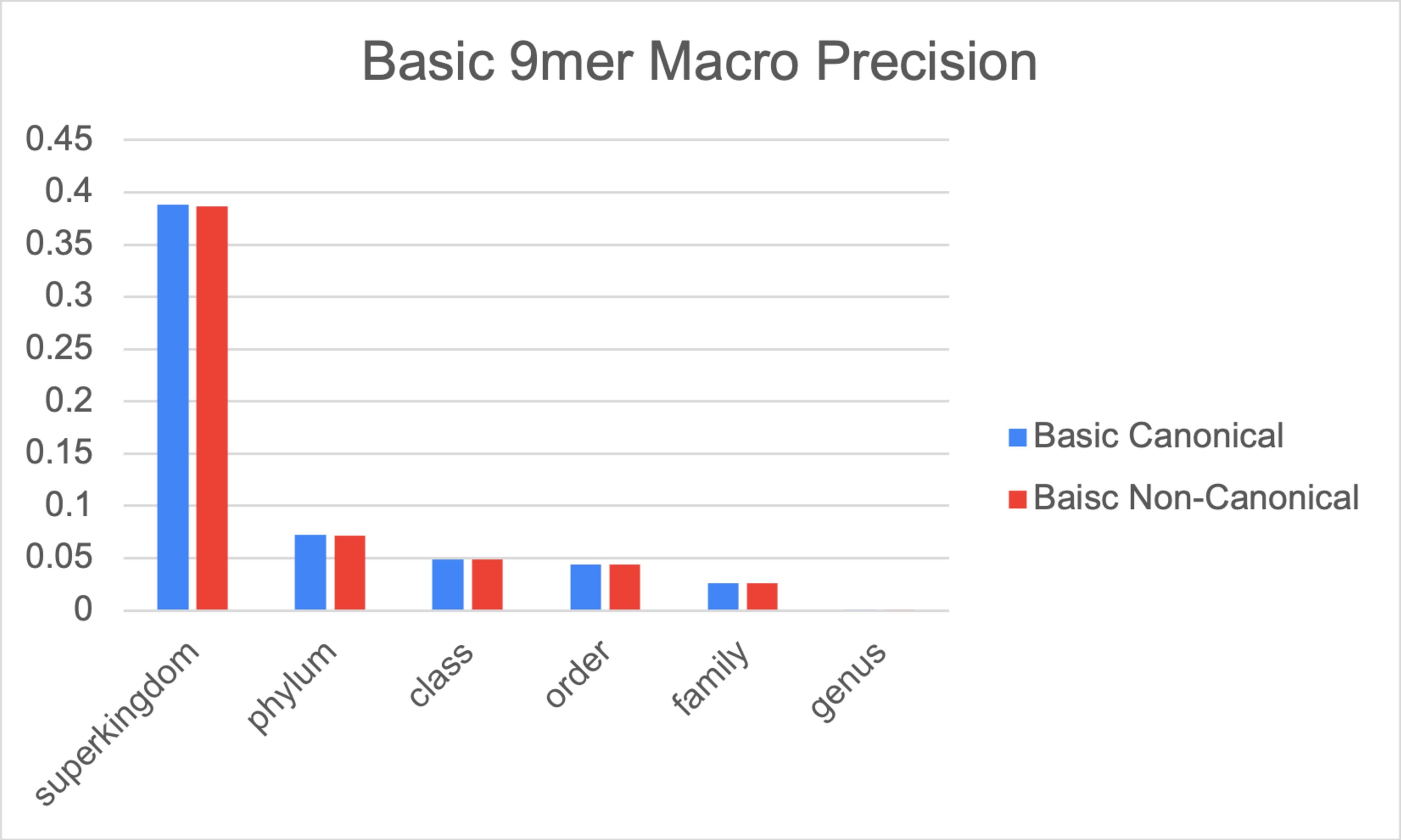

Fig. 1: Macro-Precision and Recall for Canonical vs. Non-canonical vs. taxonomic level for the Basic Database at 9mers. There is little difference in using different canonicality.

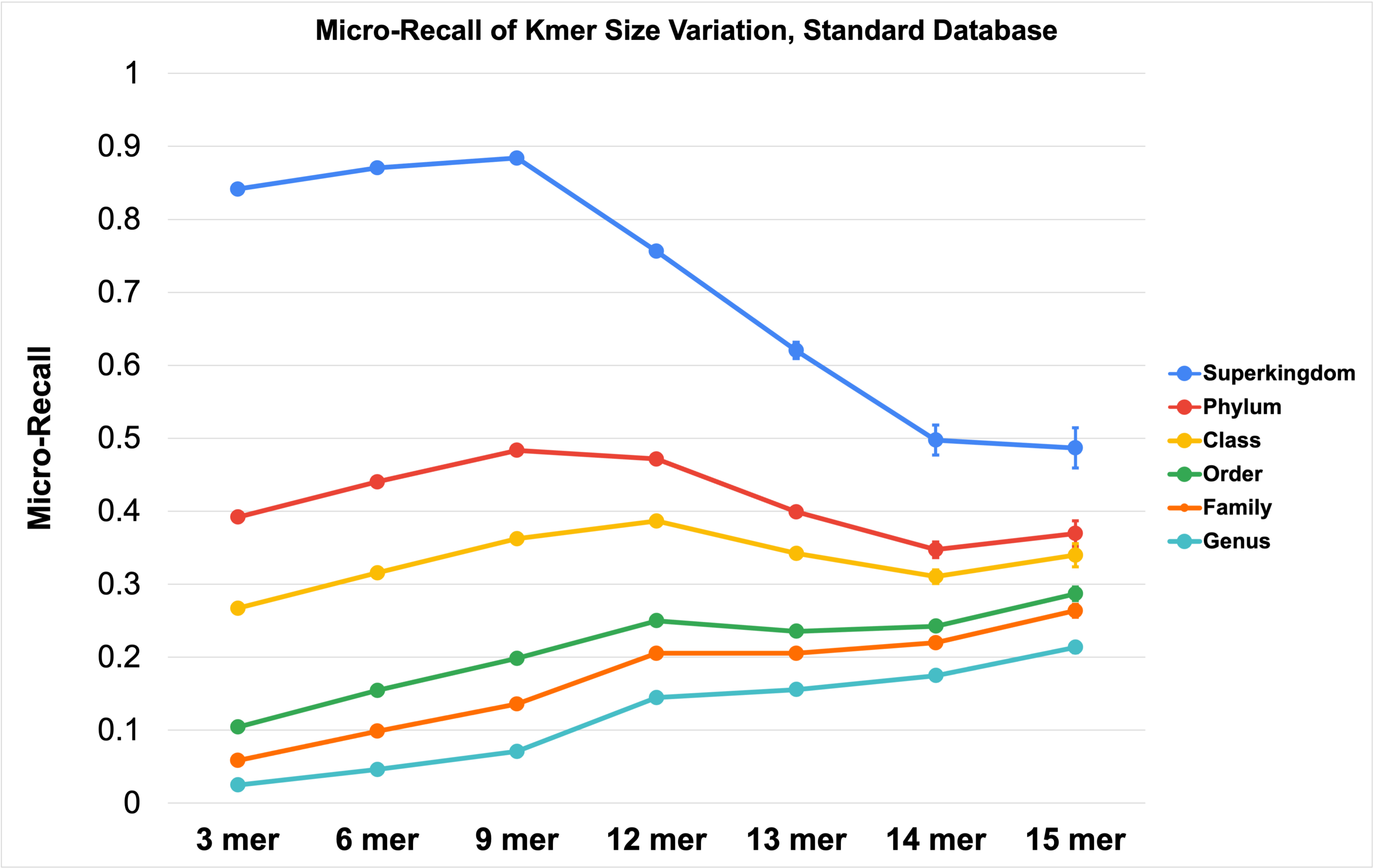

Fig. 2: Micro-Recall (Micro-Precision) vs. NBC Kmer Size for the Standard database.

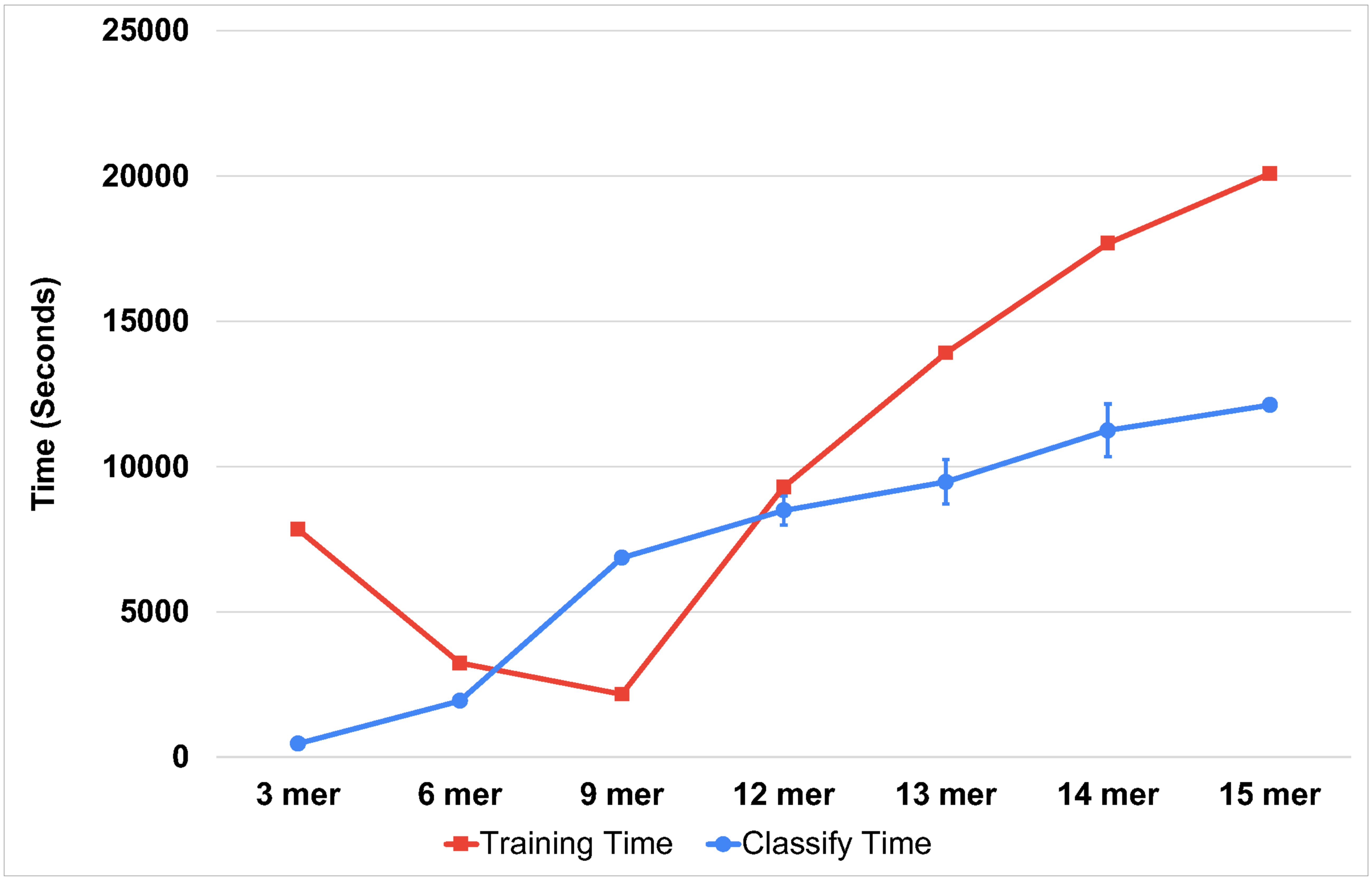

Fig. 3: Training/Classify Time vs. NBC Kmer Size for the Standard database

Average Time in Seconds

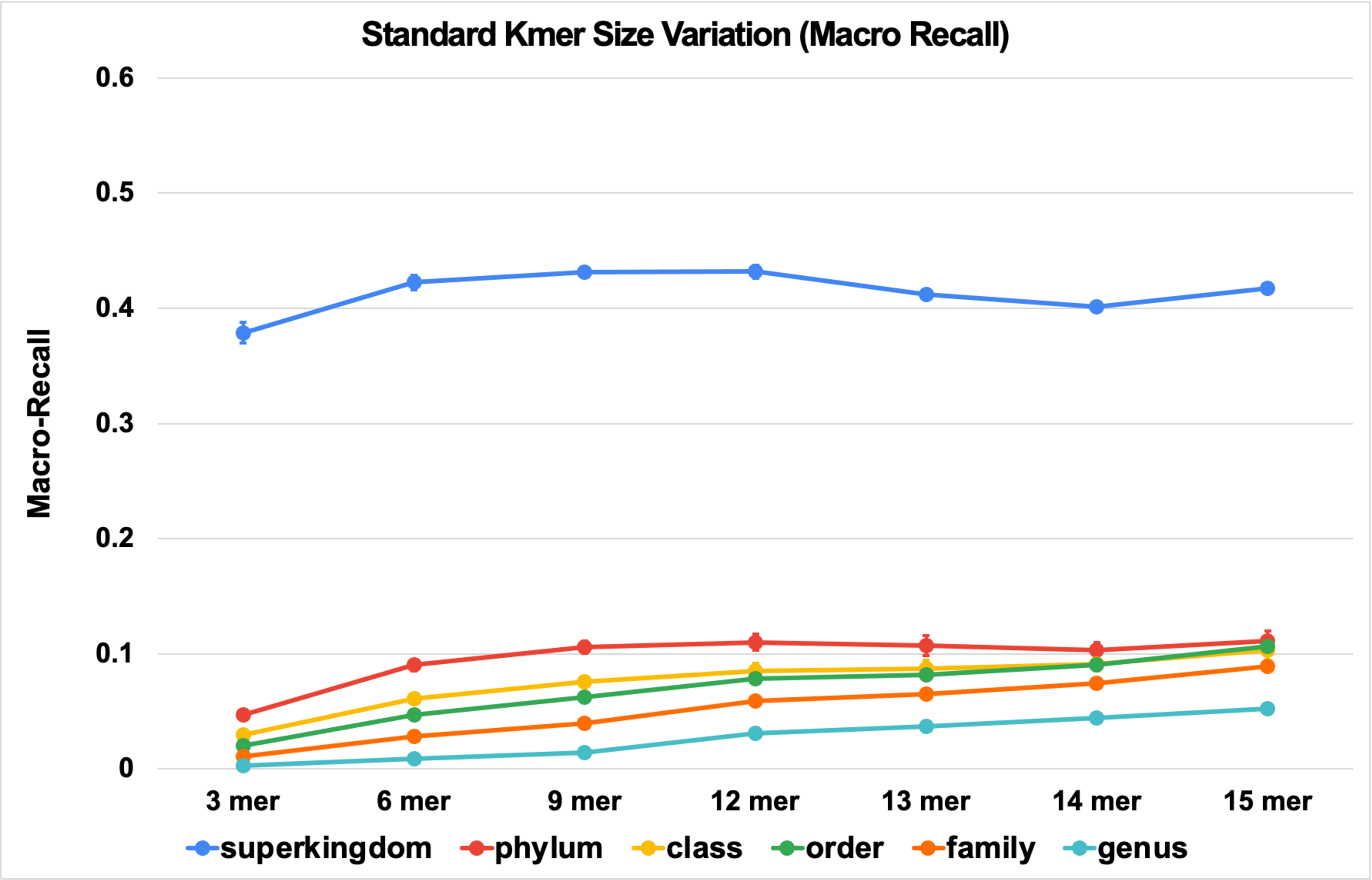

(a)

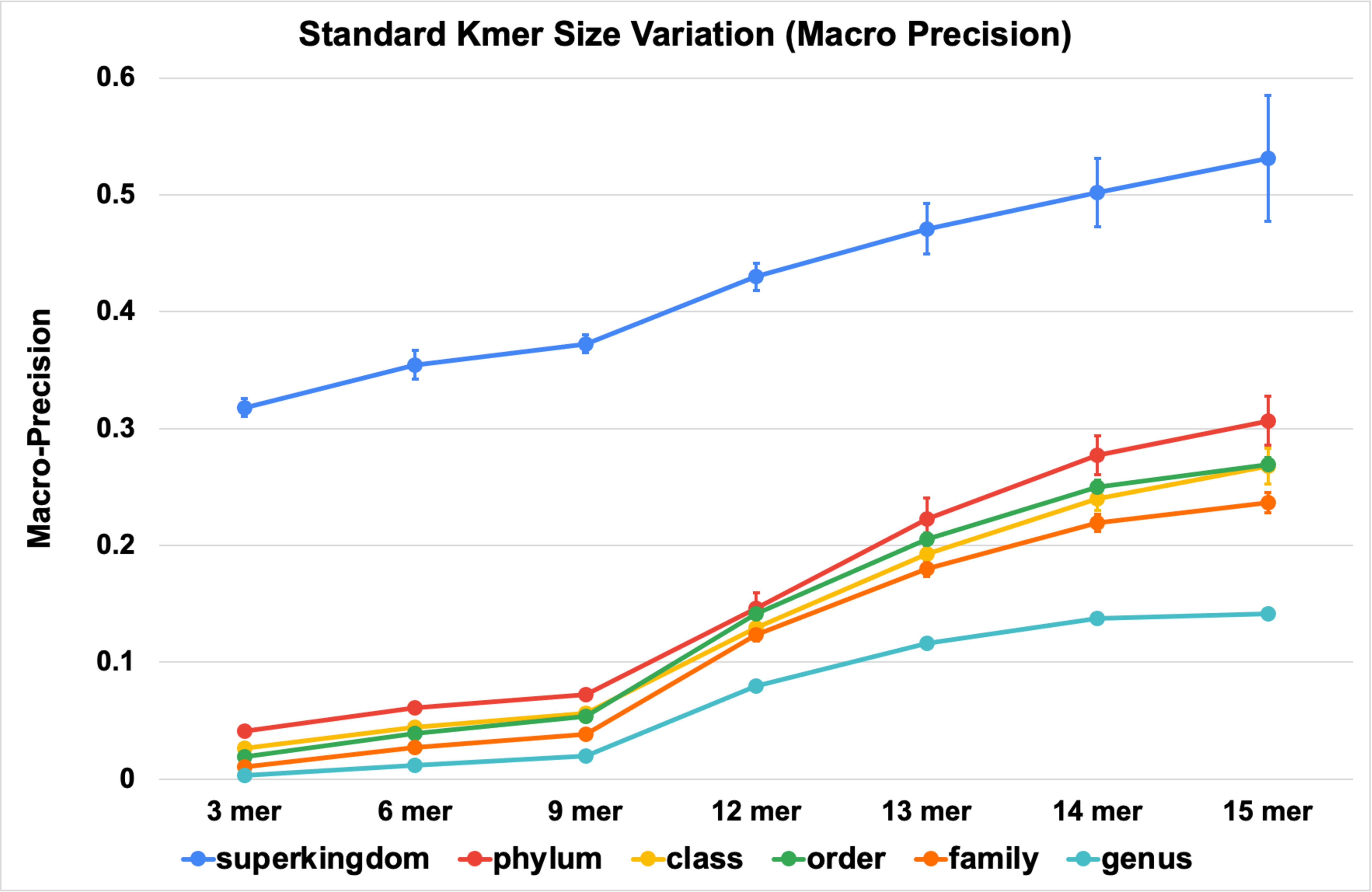

(b)

Fig. 4: Macro-Recall/Sensitivity (a) and Macro-Precision (b) vs. NBC Kmer Size for the Standard database.

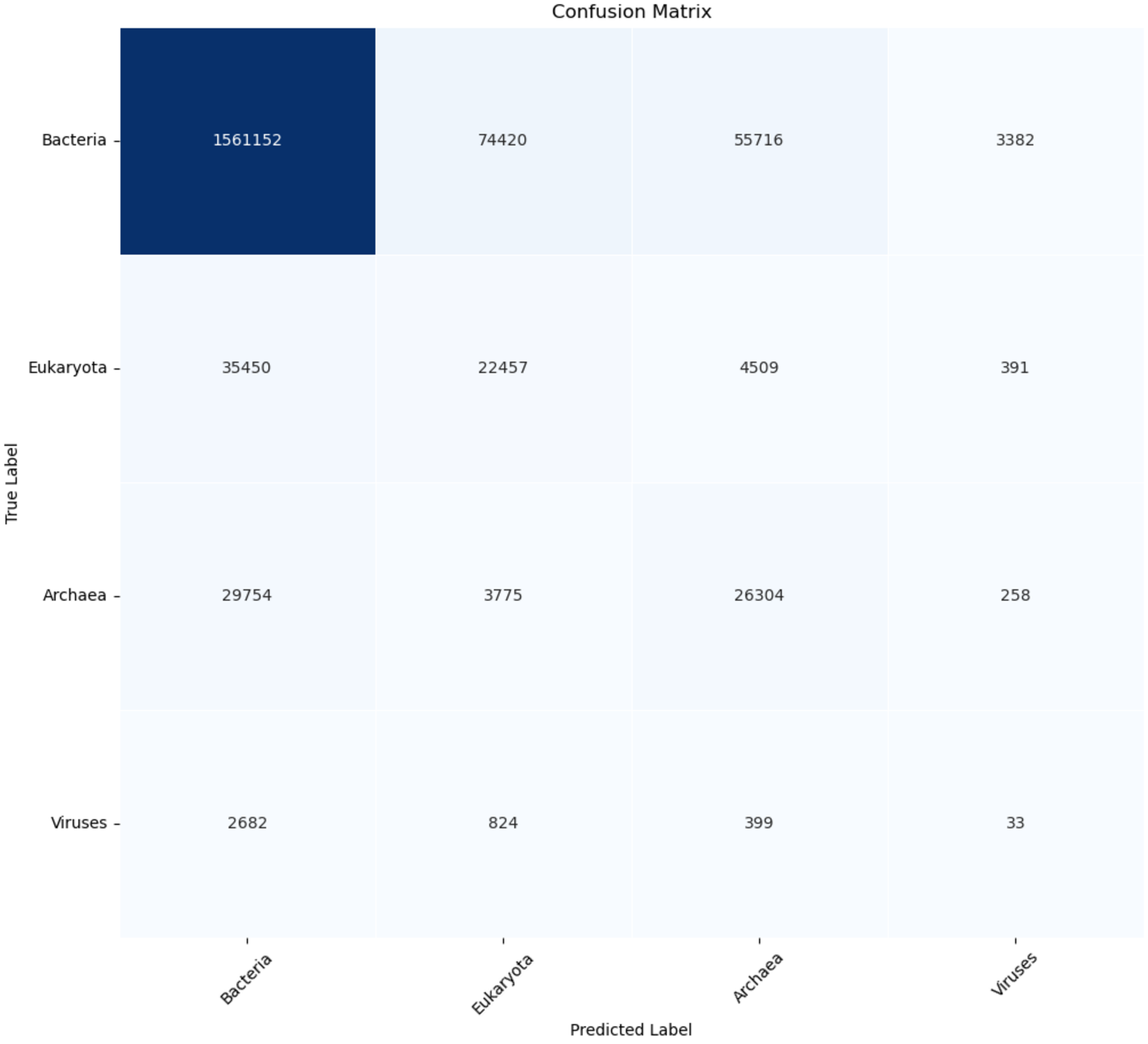

Fig. 5a: Confusion Matrix of NBC 9mer, Standard Database Superkingdom Level
Values are taken from the average of the 5 folds.
Only taxa with high concentrations in the human sample result are selected for illustration

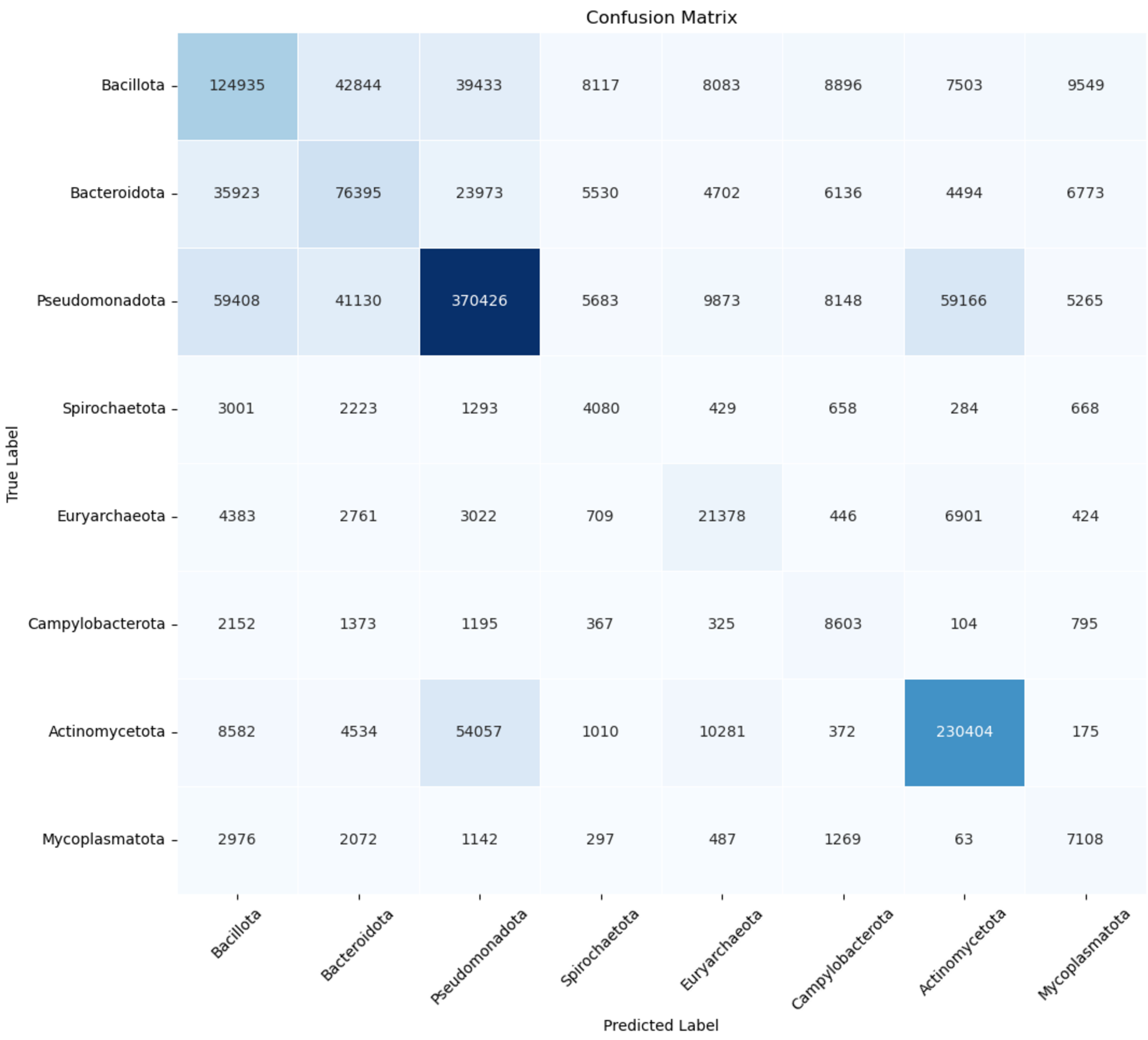

Fig. 5b: Confusion Matrix of NBC 9mer, Standard Database. Phylum Level

Values are taken from the average of the 5 folds.

Only taxa with high concentrations in the human sample result are selected for illustration

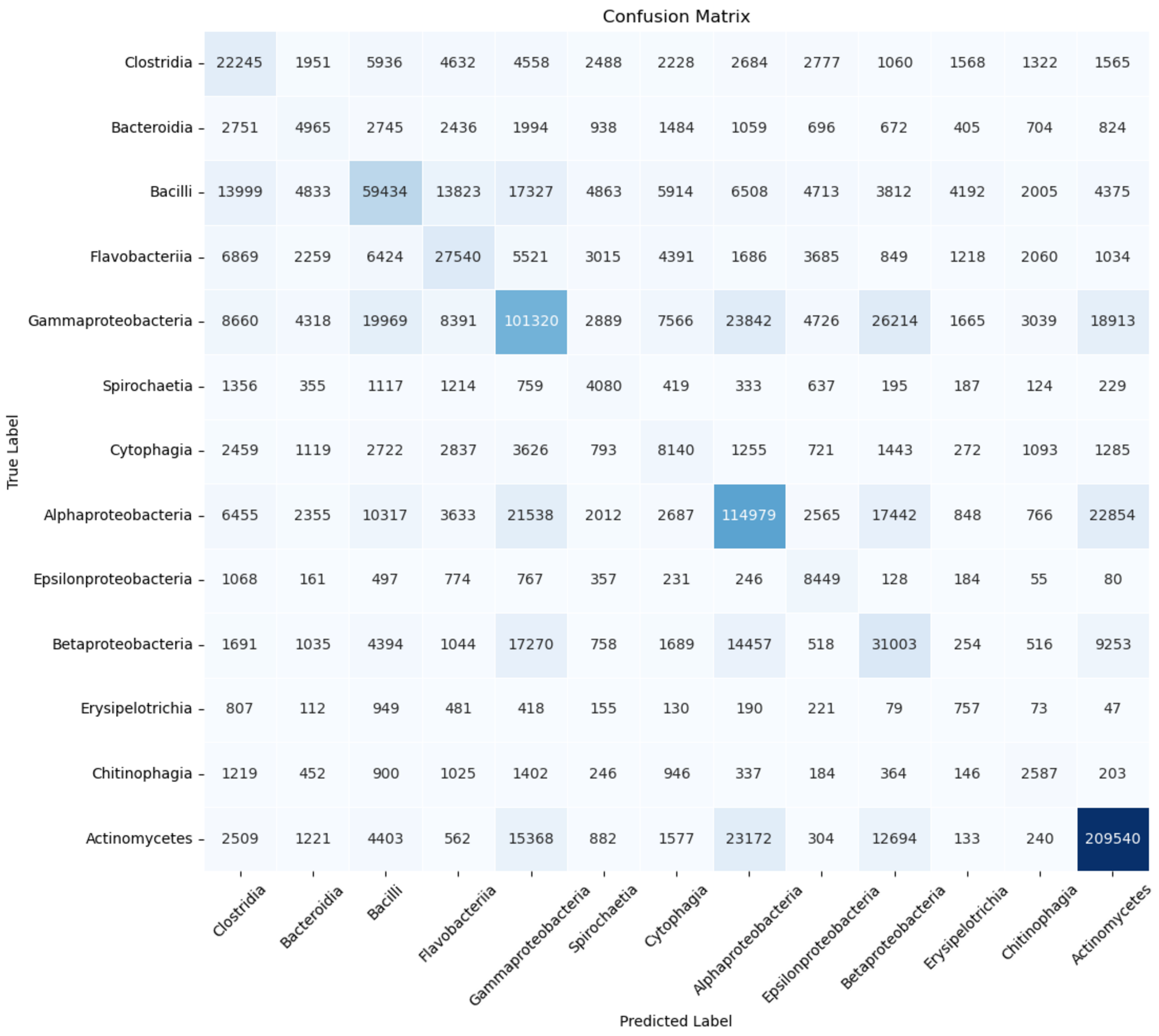

Fig. 5c: Confusion Matrix of Standard, 9 mer.
Class Level
Values are taken from the average of the 5 folds.
Only taxa with high concentration in the human sample result are selected for illustration

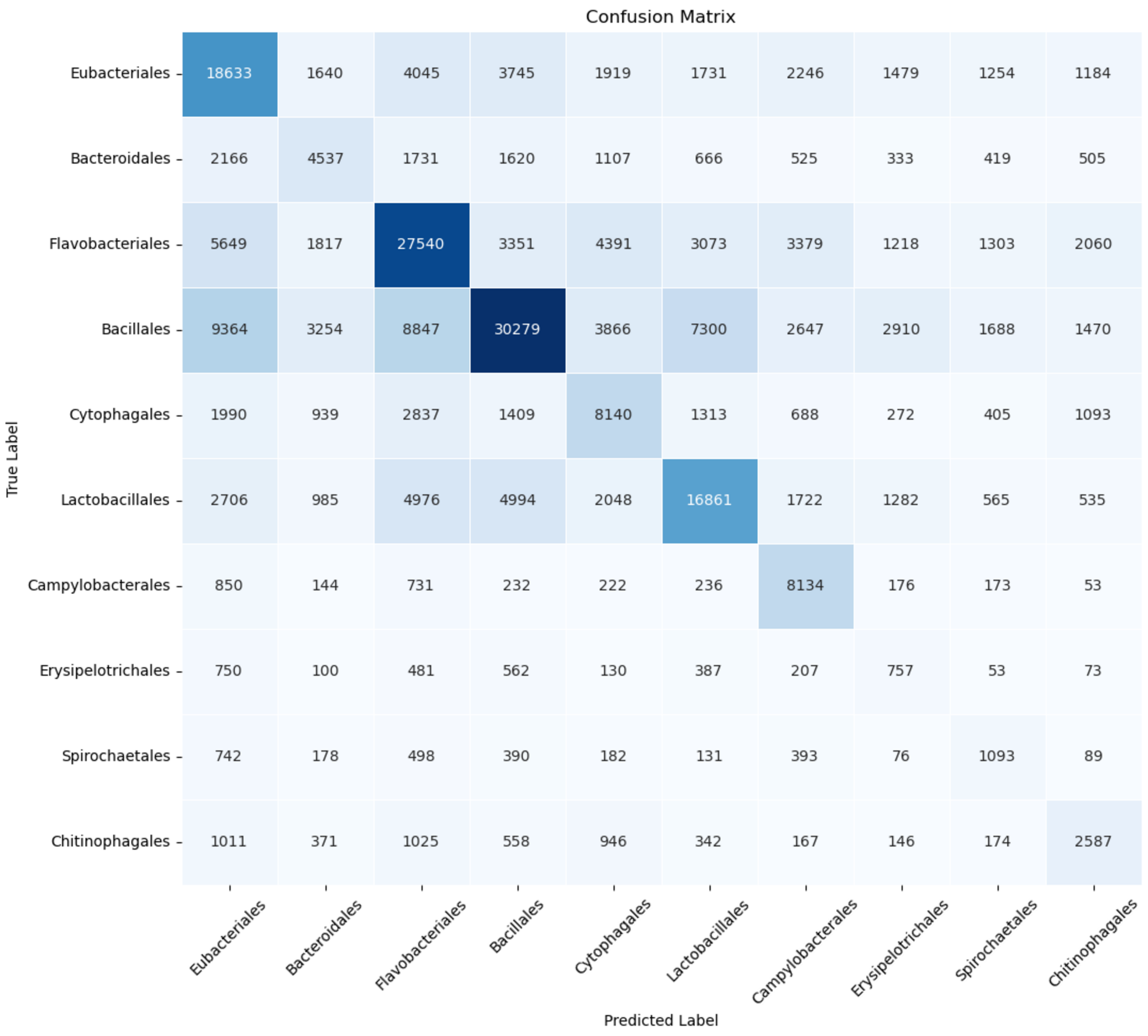

Fig. 5d: Confusion Matrix of Standard, 9 mer.
Order Level
Values are taken from the average of the 5 folds.
Only taxa with high concentration in the human sample result are selected for illustration

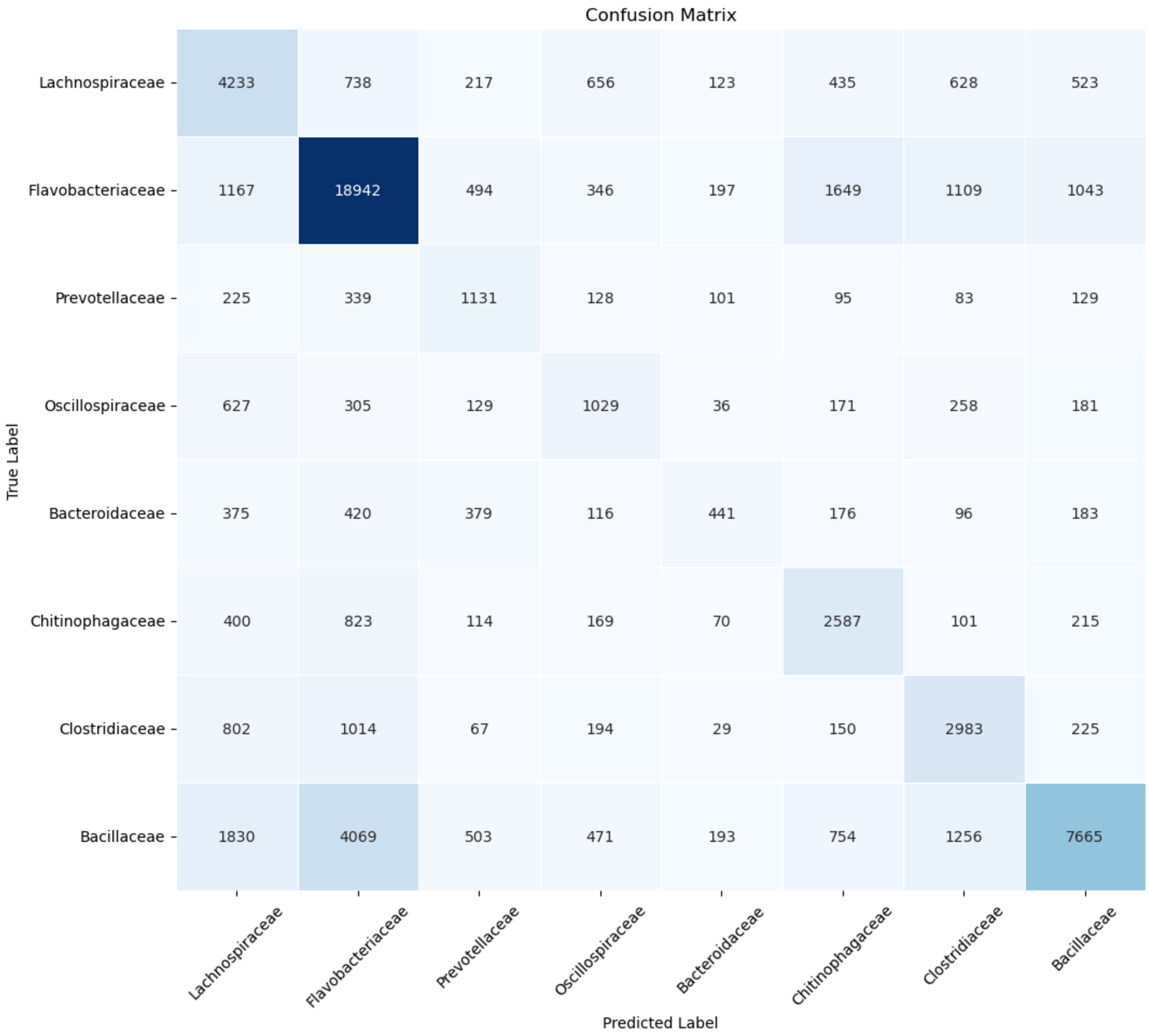

Fig. 5e: Confusion Matrix of Standard, 9 mer. Family Level Values are taken from the average of the 5 folds. Only taxa with high concentration in the human sample result are selected for illustration.

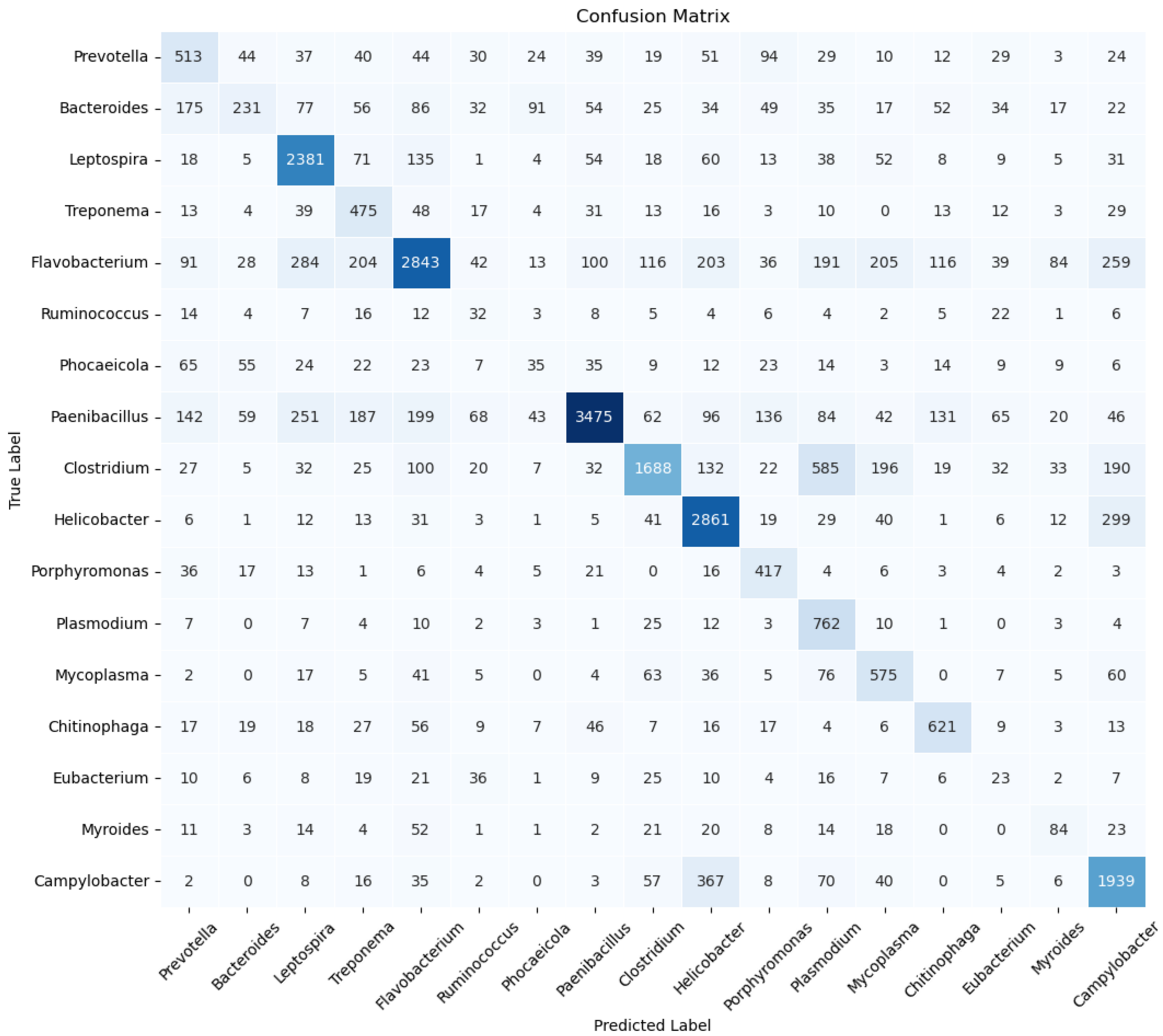

Fig. 5f: Confusion Matrix of Standard, 9 mer Genus Level
Values are taken from the average of the 5 folds.
Only the group with high concentration in the human sample result are selected

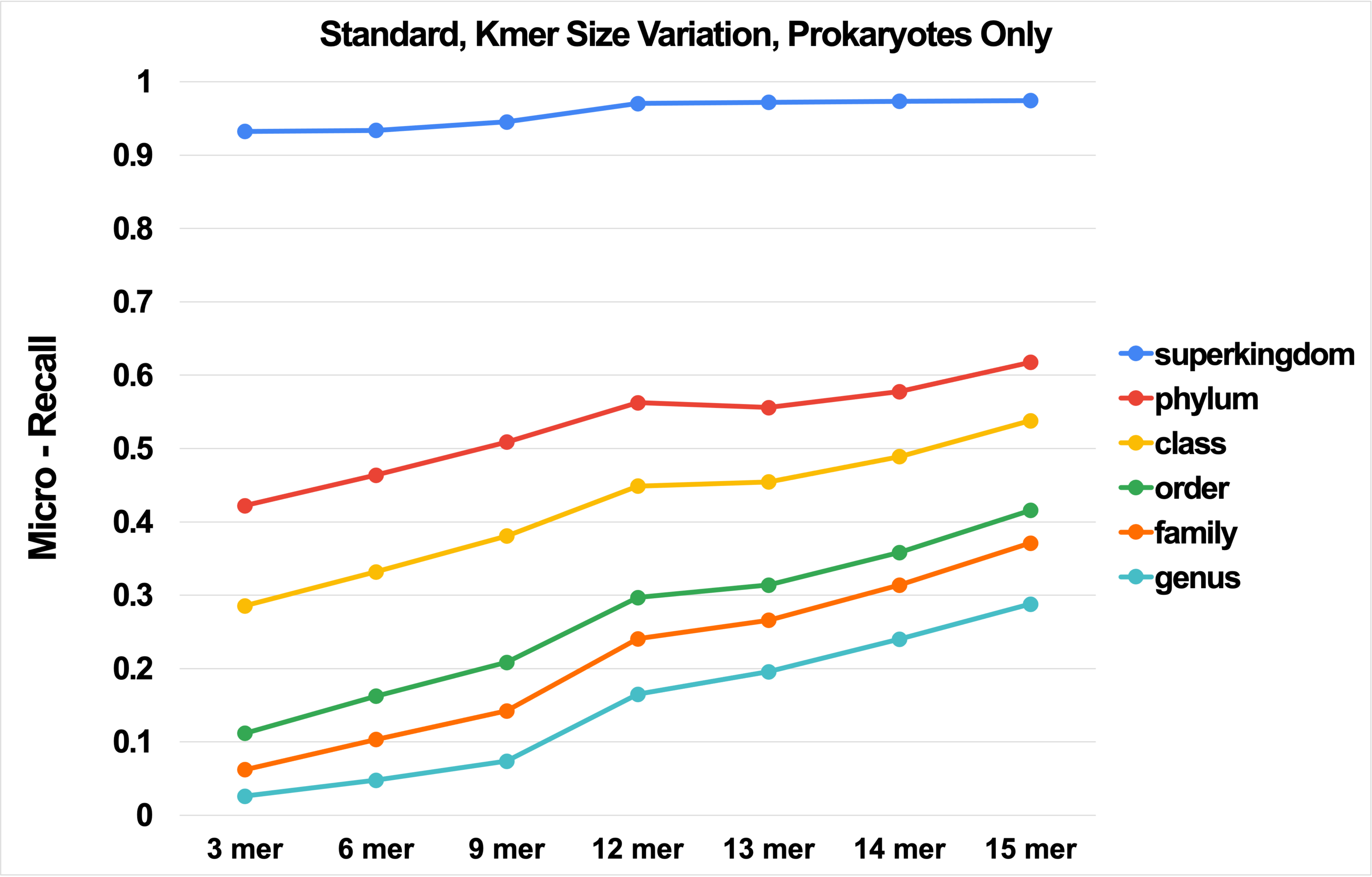

Fig. 6a: Micro-Recall (Micro-Precision) vs. NBC Kmer Size for the Standard database, Prokaryotes Only.

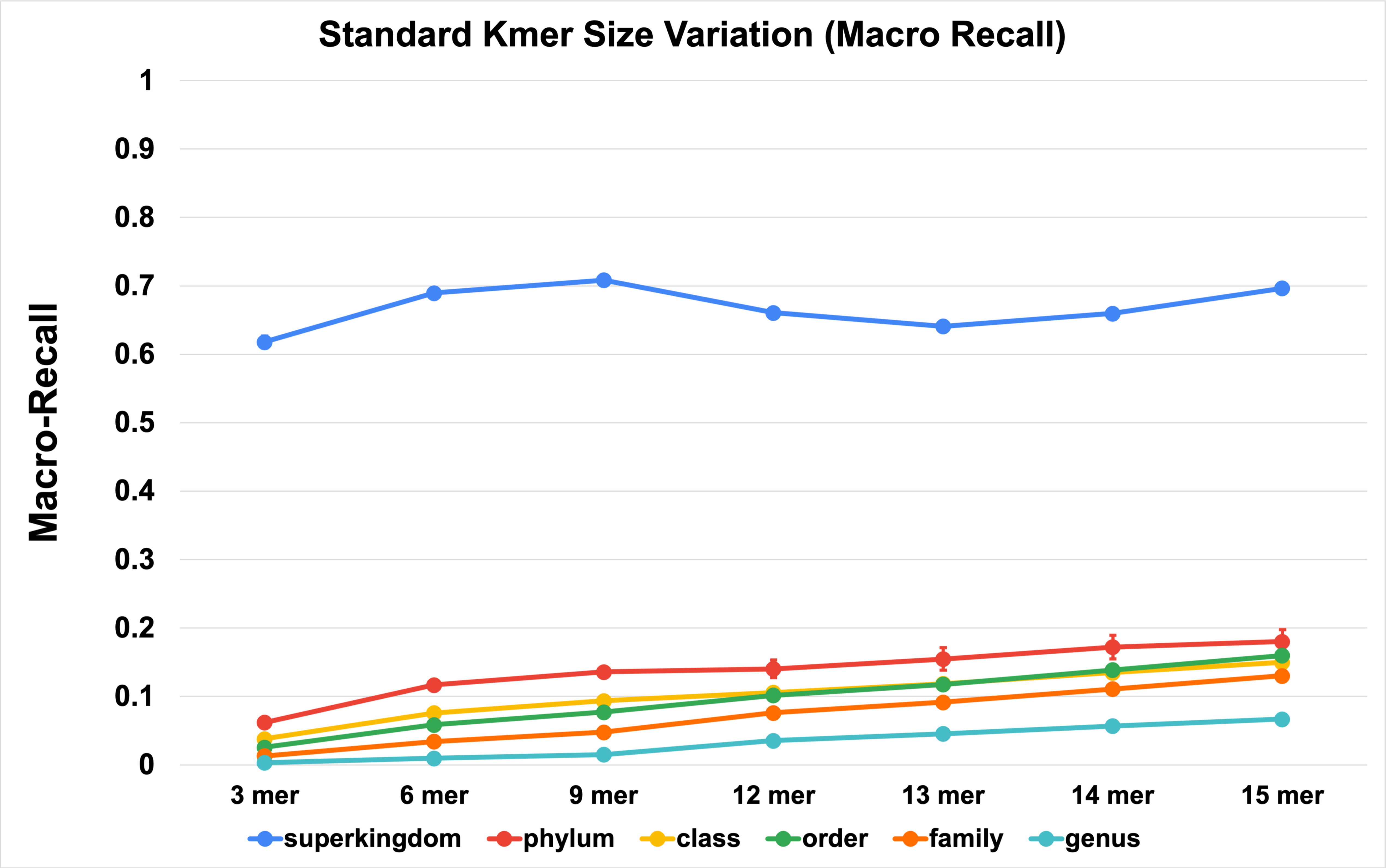

(b)

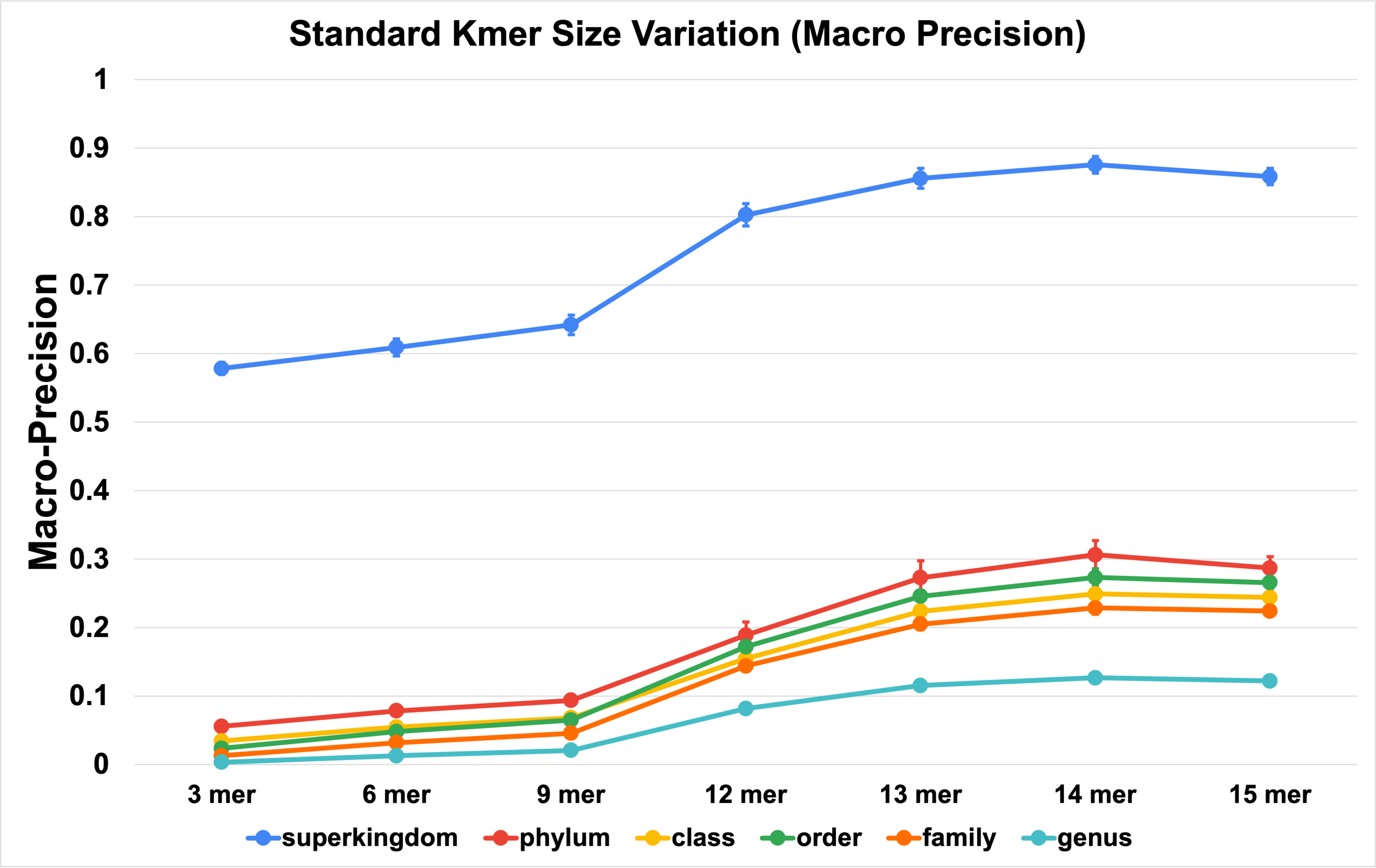

(c)

Fig. 6: Macro-Recall/Sensitivity (b) and Macro-Precision (c) vs. NBC Kmer Size for the Standard database, Prokaryotes Only

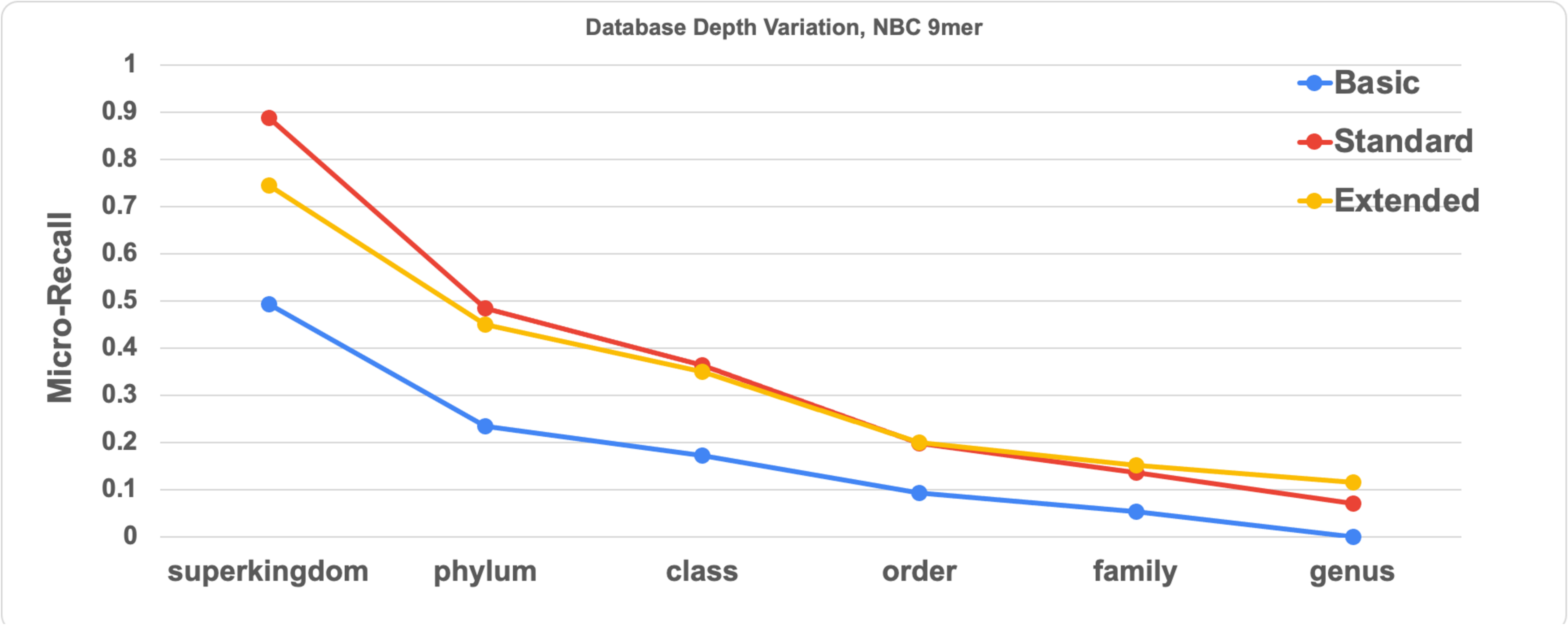

Fig. 7a: NBC-9mer Micro-Recall/Sensitivity (Micro-Precision) vs. Taxonomic Level vs. Database Depth

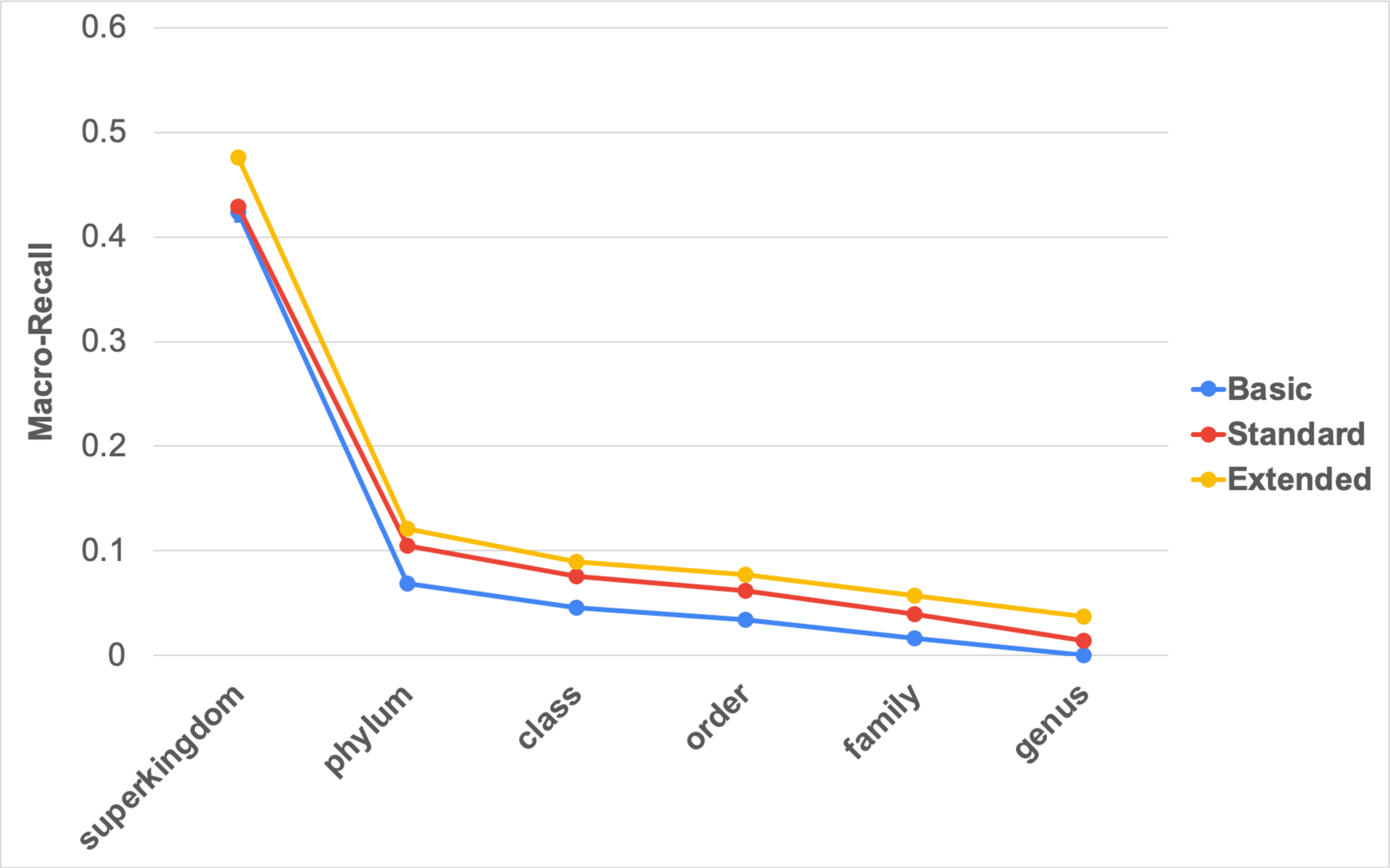

(b)

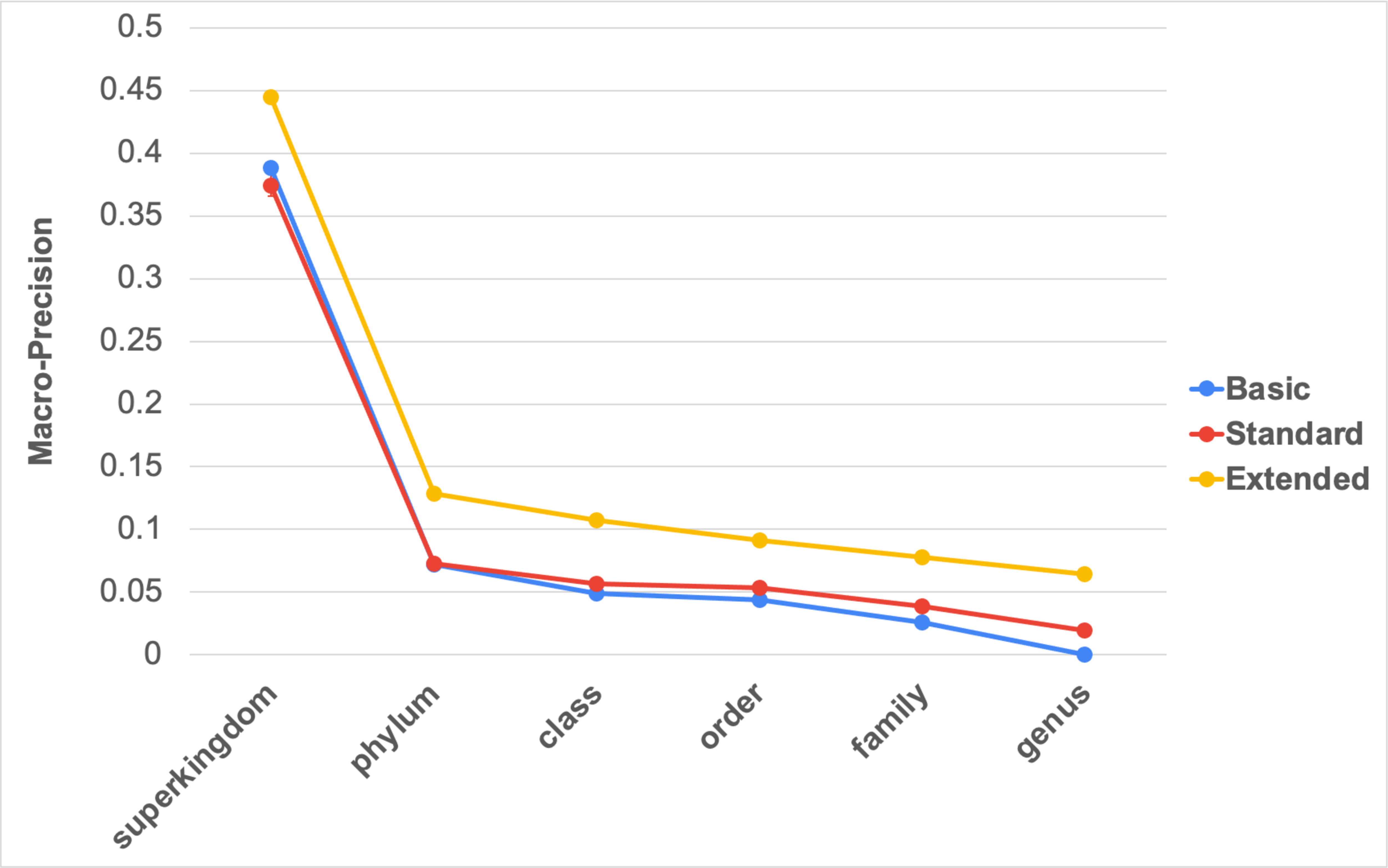

(c)

Fig. 7: NBC (for 9mers): Macro-Recall/Sensitivity (a) and Macro-Precision (b) vs. Database Depth

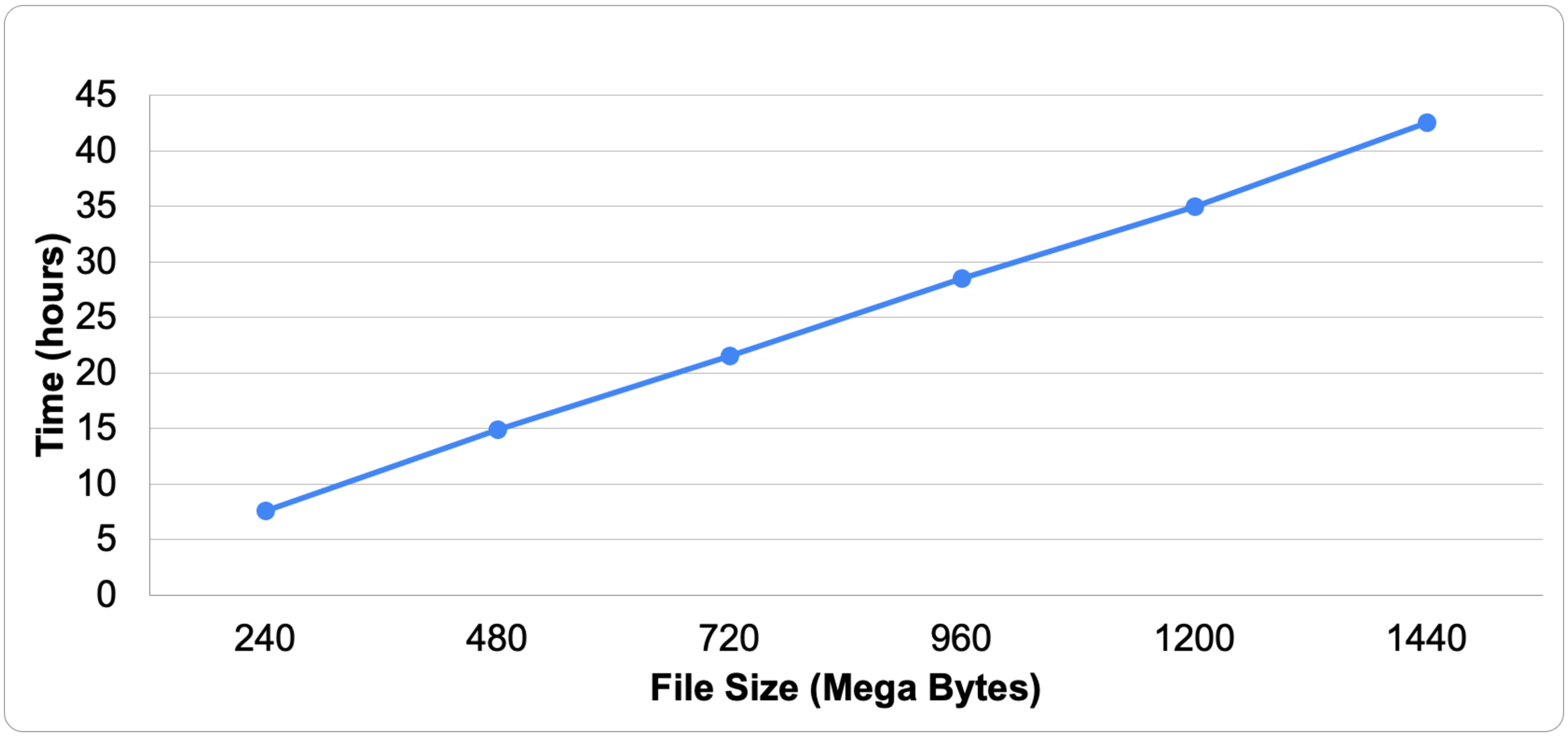

Figure 8: Time of Input File Size vs. Hours needed to Process NBC 9mers with 180GB of RAM and 48 cores.

Kraken2/Ganon2 vs. Database Depth, 1

Micro-Recall

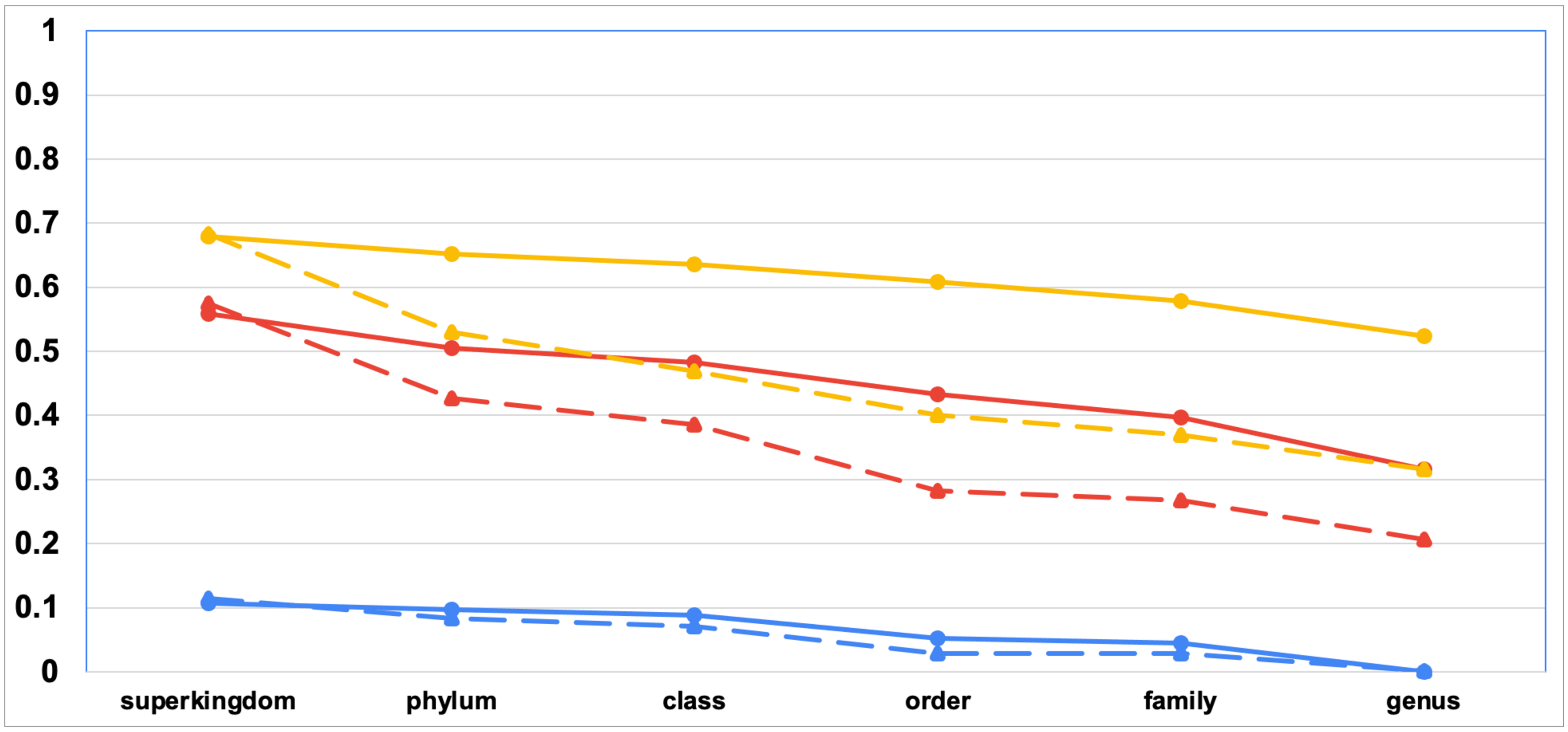

(a)

Micro-Precision

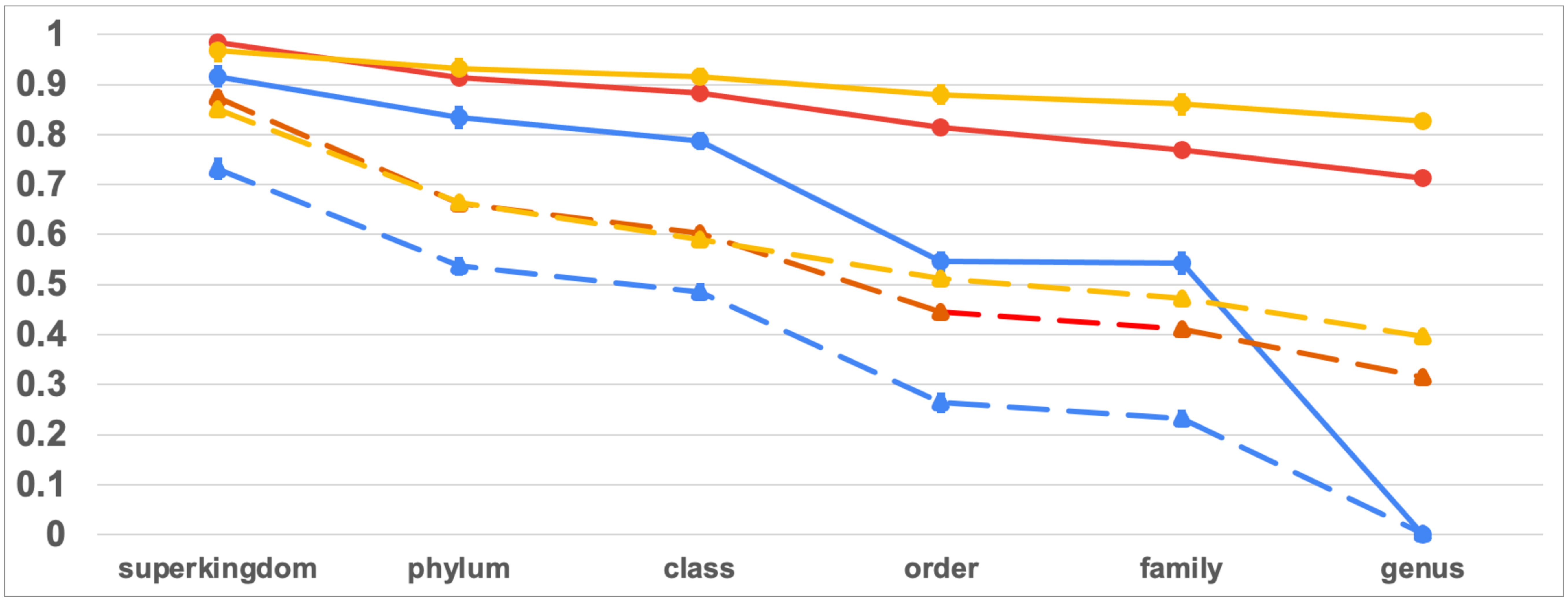

(b)

Fig. 9: Kraken2's (solid line) and Ganon2's (dashed line) Micro-Recall/Sensitivity (a) and Micro-Precision (b) vs. Database Depth (yellow - extended, red - standard, blue - basic)

Kraken2/Ganon2 vs. Database Depth, 2

Macro-Recall

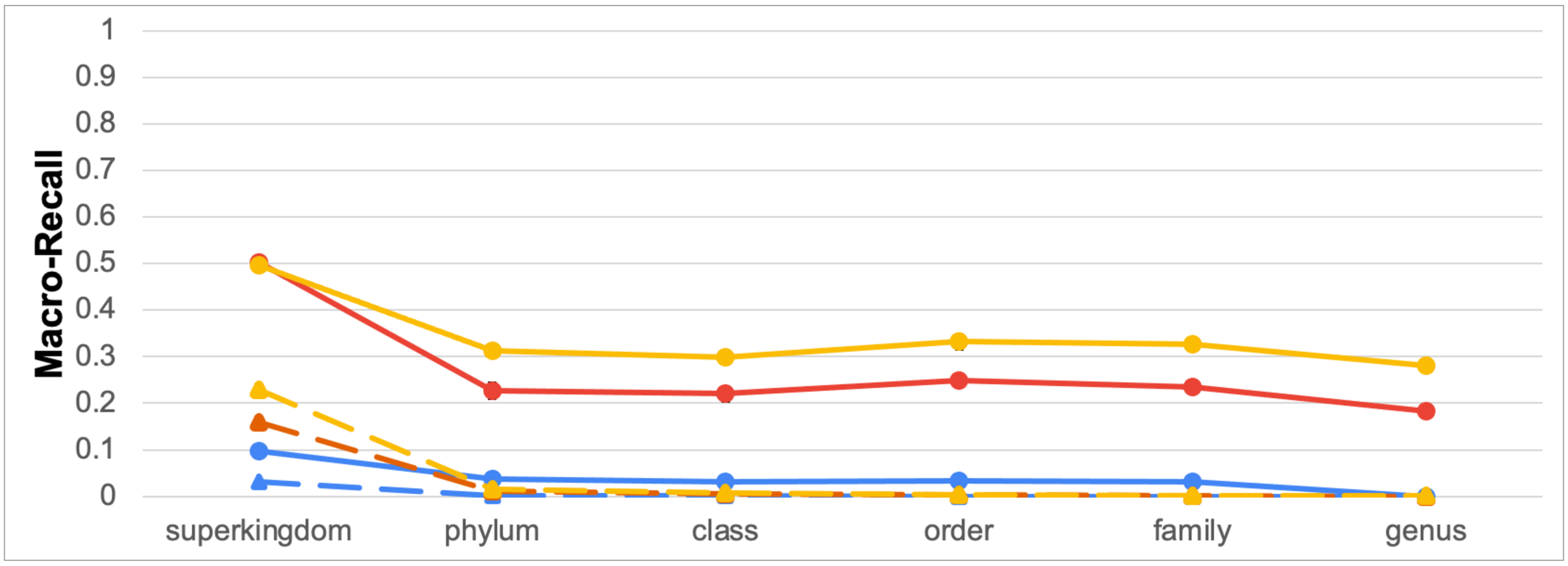

(c)

Macro-Precision

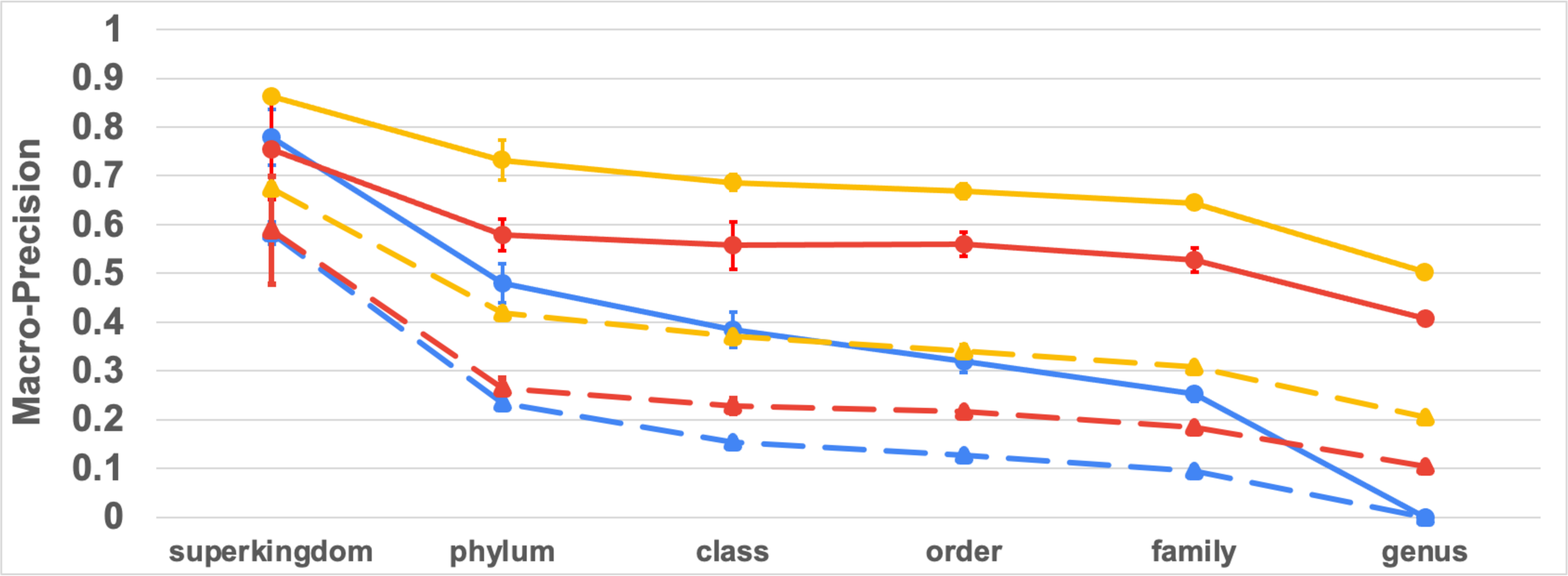

(d)

Basic Standard Extended

Fig. 9: Kraken2's (solid line) and Ganon2's (dashed line) Macro-Recall/Sensitivity (c) and Macro-Precision (d) vs. Database Depths

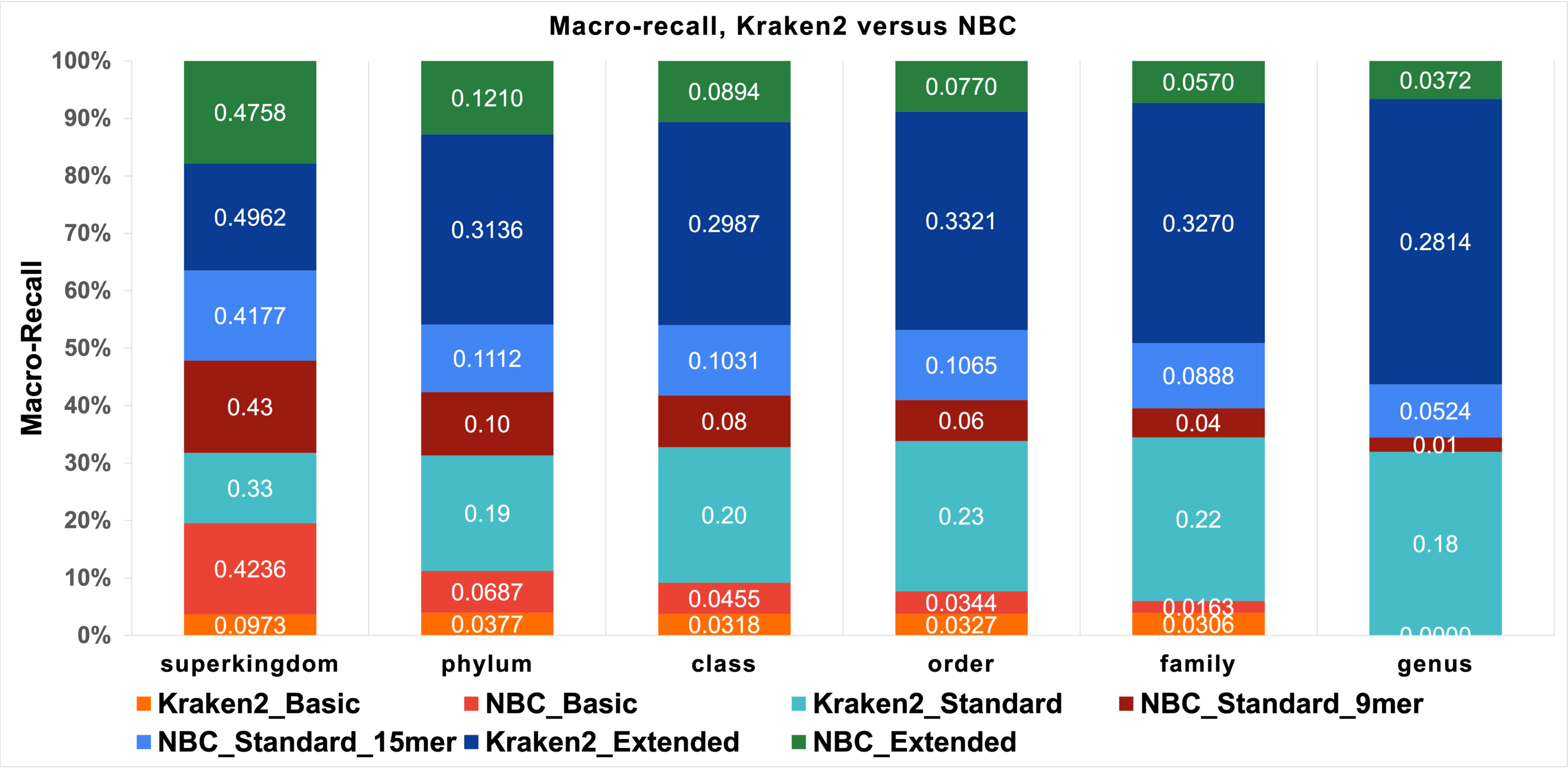

(a)

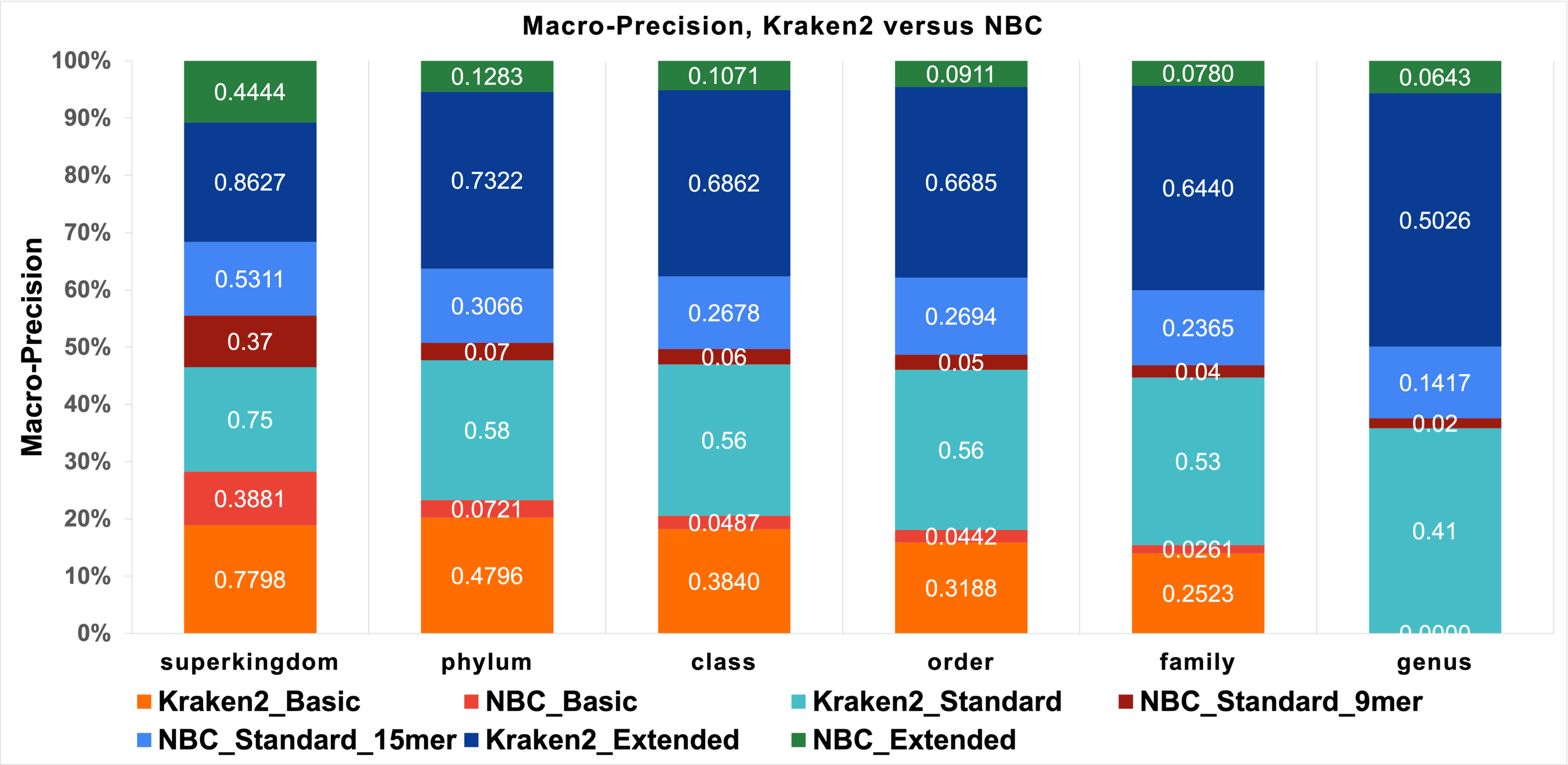

(b)

Fig. 10: Macro-Recall/Sensitivity (a) and Macro-Precision (b) NBC vs. Kraken2 vs. Database Depth. NBC performs competitively in terms of recall for the superkingdom level, but Kraken2 calls taxa with higher precision due to the confidence threshold.

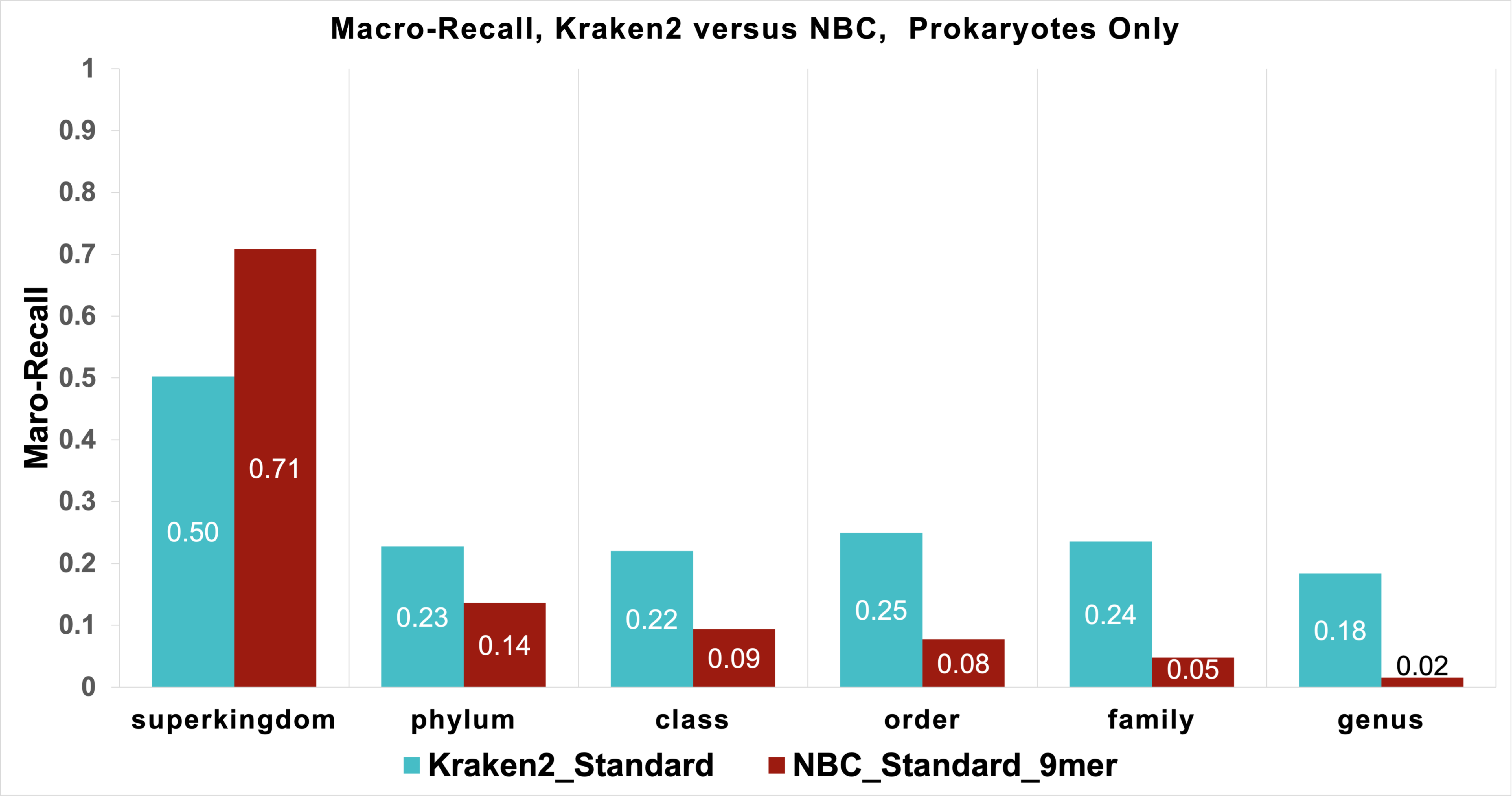

(c)

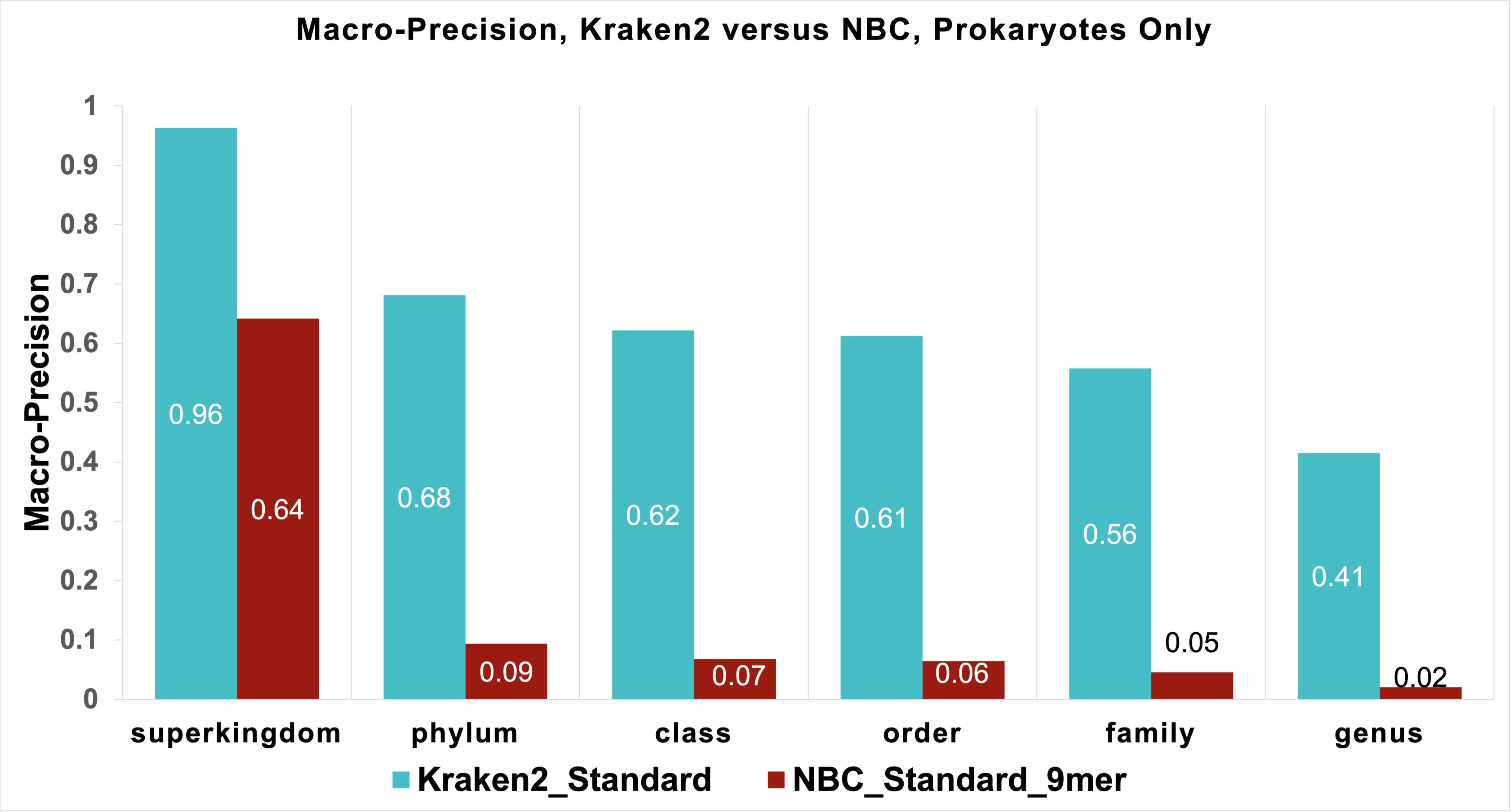

(d)

Fig. 10: Macro-Recall/Sensitivity (c) and Macro-Precision (d)
NBC vs. Kraken2 vs. Database Depths
Prokaryotes Only

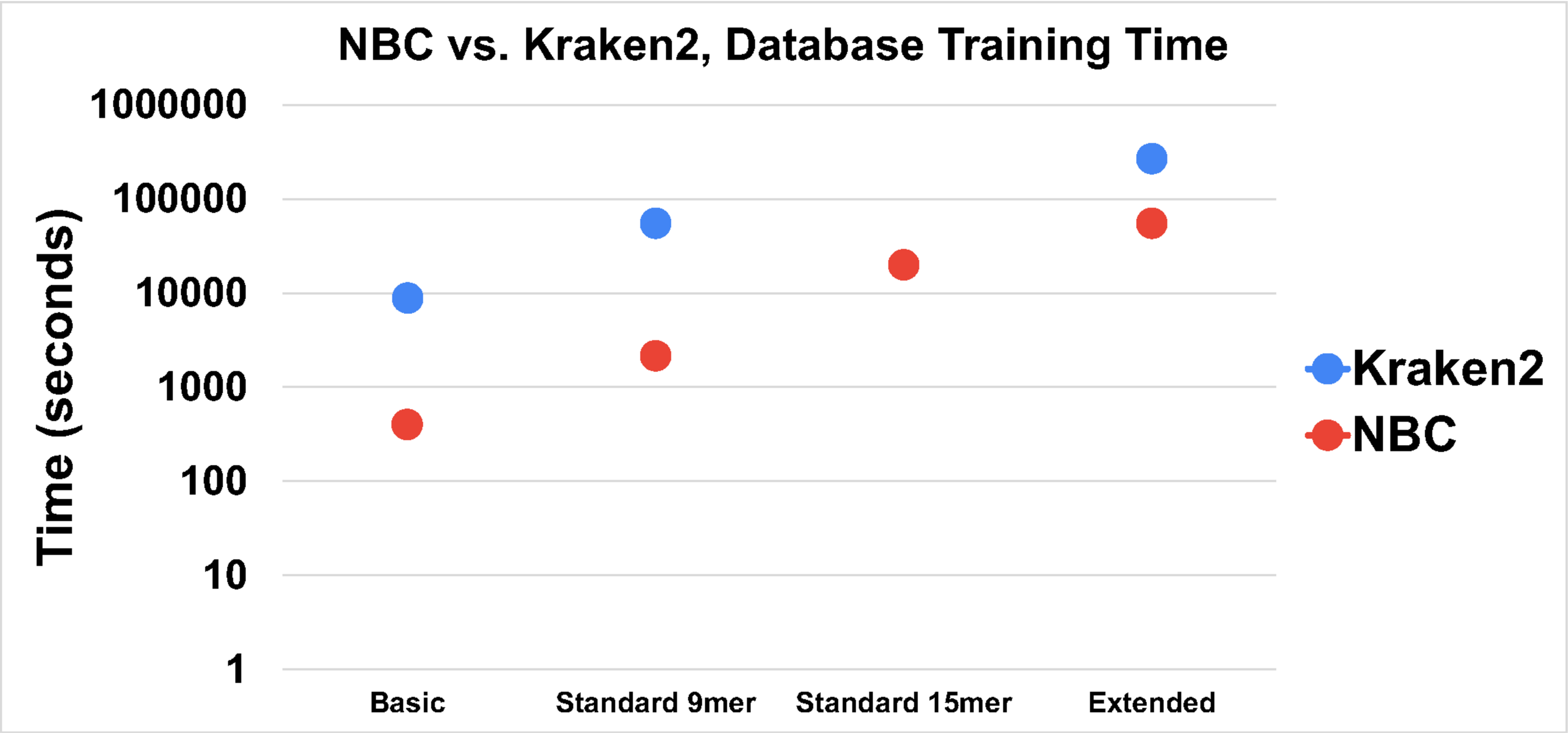

(a)

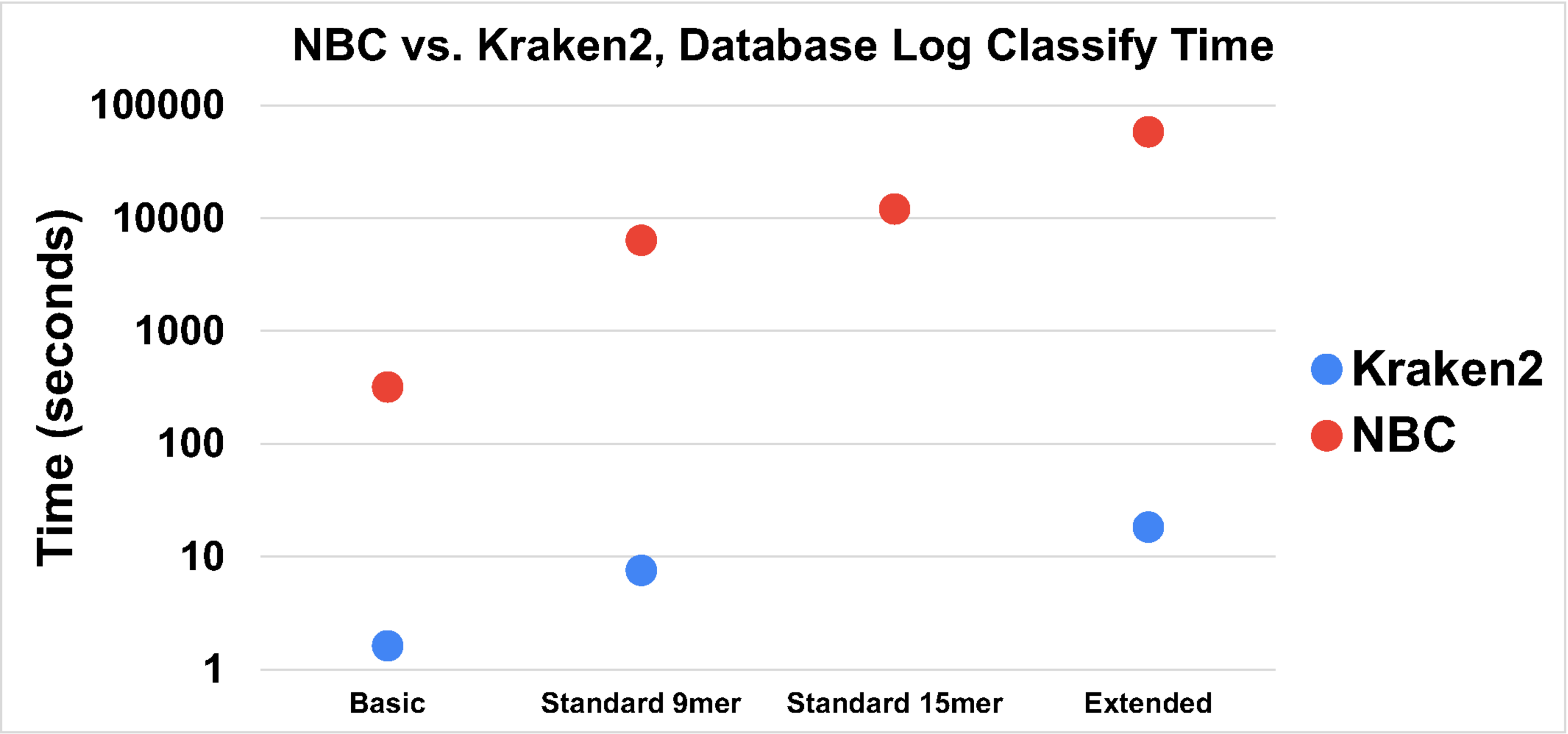

(b)

Fig. 11 Graph of the Training (a) and Testing (b) CPU times of NBC-9mers/15mers and Kraken2 (if not stated, 9mers are used for NBC).

Human Sample Composition at Superkingdom Level:

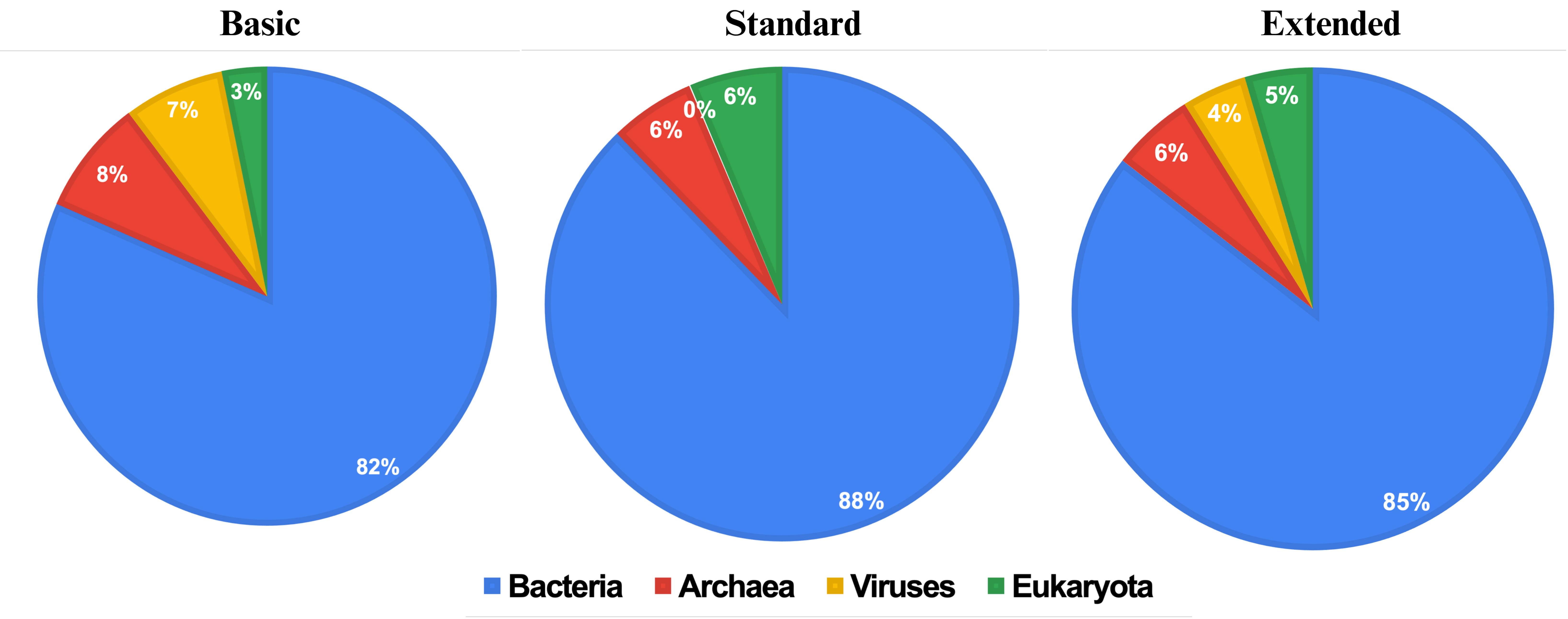

Bray Curtis Dissimilarity Score

Basic vs. Standard: 0.090

Standard vs. Extended: 0.043

Figure 12a: Superkingdoms Classified in the Human Gut Sample vs. Database Depth. The Standard Database has little viral representation while the Basic d

---

Human Sample Composition at Genus Level:

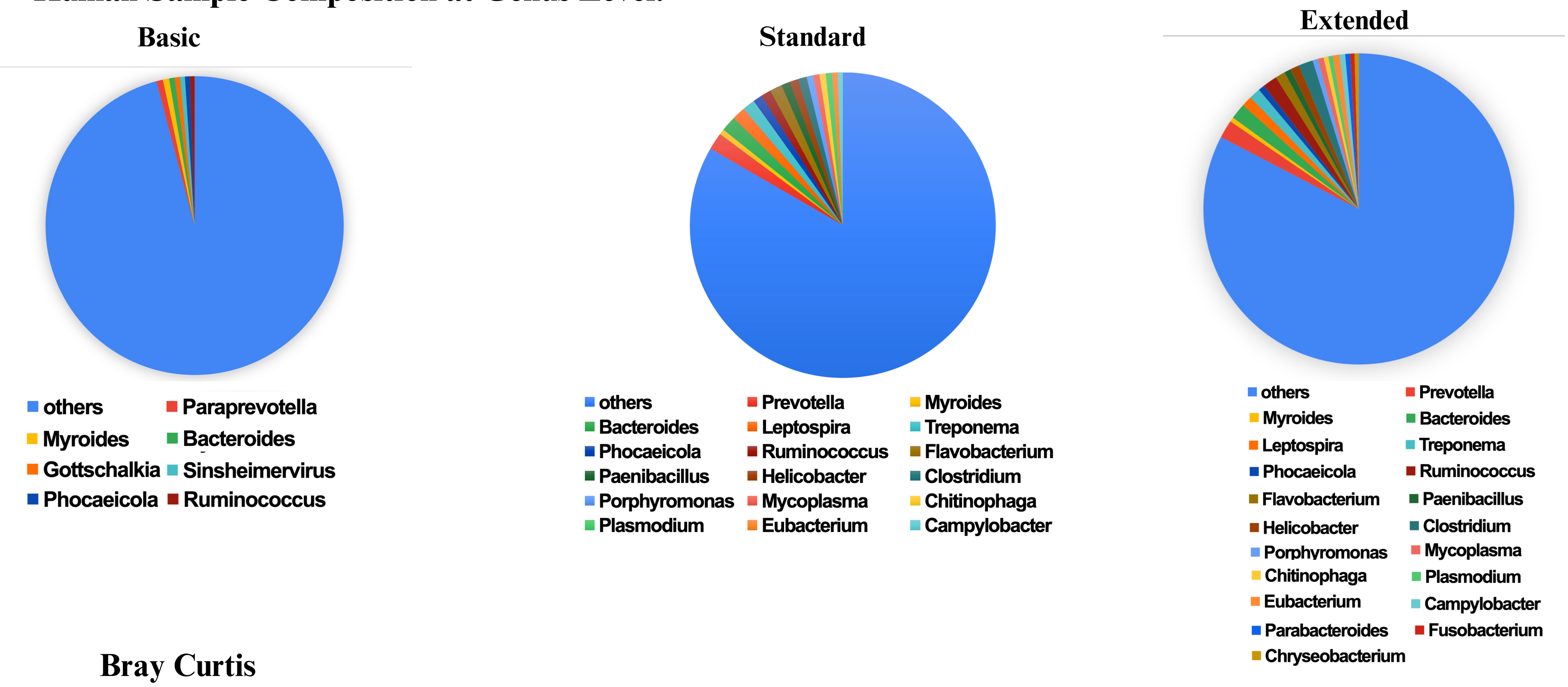

Bray Curtis Dissimilarity Score

Basic vs. Standard: 0.791

Standard vs. Extended: 0.163

Figure 12b: Genera Classified n the Human Gut Sample vs. Database Depth. The Basic database (which only has around one representative genome per genera is lacking major composition while the Standard and Extended are quite similar.
