# Supplementary material for: The Naïve Bayes classifier++ for metagenomic taxonomic classification—query evaluation
Source: Bioinformatics. 2024 Dec 19;41(1):btae743. doi: 10.1093/bioinformatics/btae743 (PMC11729721; doi:10.1093/bioinformatics/btae743)
Supplement: btae743_Supplementary_Data [file btae743_supplementary_data.zip › appendix.pdf]

Appendix

Restructuring the k-mer counting of reads to reduce I/O

A severe limit of the previous implementation required that the reads be k-mer counted before the query NBC++ code and that each read's k-mer count be stored in a separate file – such a process resulted in constant reading/writing for every read, which in turn created a heavy disk I/O load. In fact, the evaluation function in the previous NBC++ implementation did not function at all for large samples – therefore, this improvement has enabled use of the software for the large files obtained from new sequencers. Counting k-mers from reads has now been restructured to compute on-the-fly and to use the user-specified memory to determine how many to compute in a batch.

Estimating Memory Costs

Memory costs within the NBC++ system are estimated based on:

- The average memory size of a trained class: NBC++ querying memory consumption depends heavily on the k-mer size used and the length of the genome trained on. For example *Phytophthora infestans*' 9-mer file is 1.8MB and its 15-mer file is 818 MB, so 15-mers require approximately 400 × more memory at runtime.
- The average memory size per query read: usually query reads are small (∼ 100-200bp for Illumina), but occasionally, researchers may want to use contigs or even full genomes.

Allocation of Query Read Memory Buffer Size Calculations

The buffer sizes are calculated using the following formulas:

$$\begin{aligned}\text{Buffer for Query Reads} &= \text{Estimated Read Avg Length}\\ &\times \text{Thread Workload Capacity}\\ &\times \text{Number of Threads}\\ \text{Output Buffer Size} &= \text{Output Entries Memory}\\ &\times \text{Number of Threads}\end{aligned}$$

Trained Class Memory Allocation

Memory for loading classes is dynamically allocated based on the total memory specified by the user, divided by the average memory requirement for each class derived from training files, plus any additional temporary memory needed during processing. This temporary memory requirement is approximated as follows:

$$\begin{aligned}\text{Temporary Memory} &= \text{Output Data Type Size}\\ &\times \text{Threads}\\ &\times \text{Output Entries Memory}\end{aligned}$$

k-mer Smoothing may favor longer genomes that tend to have more diverse k-mer vocabularies by chance

Reconsidering the analysis from the preceding section, the natural logarithm of the vocabulary size for different k-mer lengths can be calculated as follows: for 9-mers, the upper limit to the Vocabulary value is computed using the expression $\ln\left(\frac{1}{2}\cdot 4^9\right)$, yielding approximately 11.784. For 13-mers and 15-mers, the respective expressions $\ln\left(\frac{1}{2}\cdot 4^{13}\right)$ and $\ln\left(\frac{1}{2}\cdot 4^{15}\right)$ result in values of 17.329 and 20.101, respectively. These observations delineate a trend wherein the incremental differences between the logarithmic values diminish as the k-mer length increases.

Adapting the Naive Bayes' theorem into a rearranged form yields the equation:

$$\ln\left(\frac{\text{freq}_G(kmer_i)}{N_G+V}\right) = \ln(\text{freq}_G(kmer_i)) - \ln(N_G+V)$$

Within this context, for genomes of considerable length, the propensity to include specific k-mers found within the reads escalates. As the k-mer length extends, the natural logarithm of the vocabulary size, a pivotal factor in the subtractive segment of the equation, starts to converge with this subtractive element, thereby favoring longer genomes with elevated scores.

Kraken2 add-to-lib

Because all training dataset genomes were stored in separate files, a bash script was used to sequentially apply the add-to-library function from kraken2. The runtime took a reasonable amount of time for the basic/standard datasets, but took multiple days when applied to the extended dataset. To get around this issue, the dataset was divided into 5 equivalent partitions, allowing each partition to be added simultaneously, thereby reducing the wallclock runtime to ∼24 hours from ∼100 hours. Kraken2 was then built on each library using default settings - with the only exception being the extended database, which was run using 48 cores (all other jobs were done with a single core) to reduce the runtime. The taxonomy file was downloaded early during testing, and subsequently cloned for each database.

Assembly Summary Files for the Basic, Standard, and Extended Databases

The files are in the database_assembly_summaries.zip, with the format of <database>_assembly_summary.csv.
